# Fruit and Vegetable Intake and Mental Health in Adults: A Systematic Review

**DOI:** 10.3390/nu12010115

**Published:** 2020-01-01

**Authors:** Dominika Głąbska, Dominika Guzek, Barbara Groele, Krystyna Gutkowska

**Affiliations:** 1 Department of Dietetics, Institute of Human Nutrition Sciences, Warsaw University of Life Sciences (SGGW-WULS), 159C Nowoursynowska Street, 02-787 Warsaw, Poland; barbara_groele@sggw.pl; 2 Department of Food Market and Consumer Research, Institute of Human Nutrition Sciences, Warsaw University of Life Sciences (SGGW-WULS), 159C Nowoursynowska Street, 02-787 Warsaw, Poland; dominika_guzek@sggw.pl (D.G.); krystyna_gutkowska@sggw.pl (K.G.)

**Keywords:** fruits, vegetables, juices, intake, mental health, mental disorders, depression, anxiety, well-being, quality of life

## Abstract

The role of a properly balanced diet in the prevention and treatment of mental disorders has been suggested, while vegetables and fruits have a high content of nutrients that may be of importance in the case of depressive disorders. The aim of the study was to conduct a systematic review of the observational studies analyzing association between fruit and vegetable intake and mental health in adults. The search adhered to the guidelines of Preferred Reporting Items for Systematic Reviews and Meta-Analyses (PRISMA), and the review was registered in the International Prospective Register of Systematic Reviews (PROSPERO) database (CRD42019138148). A search for peer-reviewed observational studies published until June 2019 was performed in PubMed and Web of Science databases, followed by an additional manual search for publications conducted via analyzing the references of the found studies. With respect to the intake of fruit and/or vegetable, studies that assessed the intake of fruits and/or vegetables, or their processed products (e.g., juices), as a measure expressed in grams or as the number of portions were included. Those studies that assessed the general dietary patterns were not included in the present analysis. With respect to mental health, studies that assessed all the aspects of mental health in both healthy participants and subjects with physical health problems were included, but those conducted in groups of patients with intellectual disabilities, dementia, and eating disorders were excluded. To assess bias, the Newcastle–Ottawa Scale (NOS) was applied. A total of 5911 studies were independently extracted by 2 researchers and verified if they met the inclusion criteria using a 2-stage procedure (based on the title, based on the abstract). After reviewing the full text, a total of 61 studies were selected. A narrative synthesis of the findings from the included studies was performed, which was structured around the type of outcome. The studies included mainly focused on depression and depressive symptoms, but also other characteristics ranging from general and mental well-being, quality of life, sleep quality, life satisfaction, flourishing, mood, self-efficacy, curiosity, creativity, optimism, self-esteem, stress, nervousness, or happiness, to anxiety, minor psychiatric disorders, distress, or attempted suicide, were analyzed. The most prominent results indicated that high total intake of fruits and vegetables, and some of their specific subgroups including berries, citrus, and green leafy vegetables, may promote higher levels of optimism and self-efficacy, as well as reduce the level of psychological distress, ambiguity, and cancer fatalism, and protect against depressive symptoms. However, it must be indicated that the studies included were conducted using various methodologies and in different populations, so their results were not always sufficiently comparable, which is a limitation. Taken together, it can be concluded that fruits and/or vegetables, and some of their specific subgroups, as well as processed fruits and vegetables, seems to have a positive influence on mental health, as stated in the vast majority of the included studies. Therefore, the general recommendation to consume at least 5 portions of fruit and vegetables a day may be beneficial also for mental health.

## 1. Introduction

Mental disorders (referred by the International Statistical Classification of Diseases and Related Health Problems (ICD-10) [1] as F00–F99) are indicated as a global problem by the World Health Organization (WHO). One in four individuals suffers from a mental disorder during some period of life [2], and hence, the WHO has recognized mental disorders as a health burden that should be no longer neglected [3]. Within the Comprehensive Mental Health Action Plan 2013–2020 implemented by the WHO [4], it was indicated that there is a need for, inter alia, evidence-based practice for a life course and a multidisciplinary approach to solve this problem. Taking into account the recommendation of multisectoral actions that should be taken by various specialists from the sectors of health and social services [4], the potential influence of nutrition should be included.

Some published studies have concluded that following a properly balanced diet allows maintaining a better well-being and lowers the risk of mental disorders in adolescents and students [5,6]. This finding was also confirmed by O’Neil et al. in their systematic review [7]. At the same time, a therapeutic role was indicated for the Mediterranean diet in 2 randomized controlled trials—HELFIMED [8] and SMILES [9]—for the adults suffering from depression.

However, it is not easy to assess single elements of a whole dietary pattern and recognize their effect, as each individual consumes a wide range of various products at the same time [10]. Nonetheless, some attempts have been made to identify the elements of a properly balanced diet, which are also typical for the Mediterranean diet, that may be defined as food products with beneficial effects on mental health and aiding in recovery from mental illness; these include fish and seafood, legumes, leafy greens and other vegetables, olive oil, dairy beverages, and nuts [11]. Similarly, the highest Antidepressant Food Score (AFS), calculated based on the content of 12 nutrients that are related to the prevention and treatment of depressive disorders, was given for vegetables, followed by organ meats and fruits [12].

So far, some reviews have assessed the influence of the intake of fruit and vegetable, including a meta-analysis by Liu et al. [13], which focused exclusively on the risk of depression and aroused a great deal of controversy [14,15], and a recent meta-analysis by Saghafian et al. [16], which also focused exclusively on the risk of depression. In addition, a systematic review of prospective research, analyzing the effect on psychological health, was performed by Tuck et al. [17], but it focused exclusively on vegetables. A review by Rooney et al. [18] analyzed the role of fruits and vegetables in the broad aspects of psychological well-being, but it was not a systematic review and the authors emphasized the need for a more exhaustive synthesis of studies in the form of a systematic review.

Fruit and vegetables are beneficial for general health and the recent studies indicate that they may be even more important than it was previously supposed and that to obtain prevention of cardiovascular disease, cancer, and premature mortality even the higher intake than the generally-recommended 400 g is needed [19]. However, the mechanism of their influence on mental health is still unknown, while a number of possible factors that may contribute to the positive impact are indicated [18]. Among them, there are specific nutrients, which are known as such that may be related to mental health and for which fruit and vegetables are indicated as a valuable sources in diet, such as complex carbohydrates [20] and fiber [21], being associated with glycemic index [22], C vitamin [23], B vitamins [24], carotenoids [25], potassium [26], and polyphenols [27]. The other explanations are associated with either possibility of reverse mechanism (higher level of mental health may promote better diet, including higher fruit and vegetables intake) [28], or psychological explanation (following better diet, including higher fruit and vegetables intake may promote more positive emotions and better mental health) [29].

Taking this into account, the present study aimed to conduct a systematic review of the observational studies analyzing the association between the intake of fruit and vegetables and the broad aspects of mental health in adults.

## 2. Materials and Methods

### 2.1. Study Design

The search used Preferred Reporting Items for Systematic Reviews and Meta-Analyses (PRISMA) guidelines [30] and the review was registered in International Prospective Register of Systematic Reviews (PROSPERO) database (CRD42019138148). A systematic review of peer-reviewed observational studies published until June 2019 was performed in PubMed and Web of Science databases with the additional manual search conducted via references of found studies.

### 2.2. Inclusion/Exclusion Criteria

The present systematic review analyzed the association between the intake of fruit and/or vegetable and mental health in adults. The studies included were those that assessed the intake of fruit or vegetables (conducted using various methods) to specify the amount consumed (supposed reason), together with an assessment of mental health (various factors, both self-assessed and diagnosed) (supposed consequence) and an analysis of the association between the indicated factors. The studies which analyzed the reverse association (assessment of the intake of fruit or vegetables as influenced by mental health) were not included, but those that did not define the supposed reason and consequence and considered only the intake of fruit or vegetables and mental health as coexisting factors were included.

With respect to the intake of fruit and/or vegetables, studies that assessed the intake of fruits and/or vegetables, or their processed products (e.g., juices), either as a measure expressed in grams or as the number of portions, were included. Studies involving the analysis of highly processed products (e.g., jam, beverages other than juices) were excluded. Only those studies presenting the observations of habitual intake, with no short-term interventions, were included. The studies that did not assess single products (intake of a specific fruit or vegetable) or groups (total intake of fruits and/or vegetables) but assessed the general dietary patterns were analyzed; however, they were not included in the present analysis if there was no separate assessment of the intake of fruit or vegetables.

With respect to mental health, studies that assessed all the aspects of mental health in both healthy participants and those with physical health problems were included. However, studies conducted in groups of patients with (1) intellectual disabilities, (2) dementia, and (3) eating disorders were excluded. Only studies conducted in humans and especially adults were included.

The included studies presented the research conducted in all the countries, independent of the location and income, but only those published in English, in peer-reviewed journals were taken into account.

### 2.3. Search Strategy

The search for studies was performed in PubMed and Web of Science databases; the strategy applied for electronic search in both databases is presented in Supplementary Table S1. In addition, a manual search for publications was conducted via analyzing the references of the found studies. The search focused on gathering observational studies published until June 2019.

Studies were independently extracted by 2 researchers and screened based on the inclusion and exclusion criteria using a 2-stage procedure: (1) Studies were verified based on the title; and (2) studies included based on the title were verified based on the abstract. Any disagreement between the two researchers over including a particular study was resolved through discussion with a third researcher. The full texts of these potentially eligible studies were retrieved (by asking the corresponding author for the full text, if needed) and independently assessed for eligibility by the two researchers. Again, any disagreement between them over the eligibility of a particular study was resolved through discussion with a third researcher.

**Figure 1:**
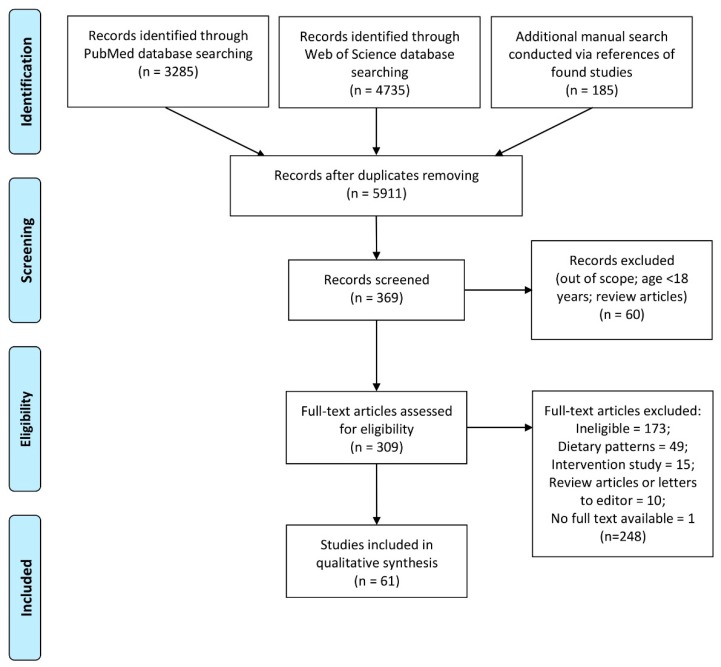
Procedure of identification, screening, eligibility assessment, and inclusion within the conducted systematic review.

The procedure of identification, screening, assessment of eligibility, and inclusion is presented in Figure 1.

### 2.4. Data Extraction

Data extraction was carried out independently by two researchers. Any disagreement between them over specific data was resolved through discussion with a third researcher. Missing data were requested from the authors of the studies, if possible, while the total number of 46 individual e-mails have been sent to corresponding authors of the included studies, as well as to first authors (if the first author was not corresponding one, but his e-mail address was provided in the full text of article). For 6 studies, authors not only answered, they also provided the requested data (in tables those data are referred as provided by authors on request).

Data extracted from the studies included the following: General details of the study (author, study design), observation (country/location, study group, time), participants (number of participants, gender proportions, age, inclusion criteria, exclusion criteria), exposure (method of assessment, measure of fruit and vegetable, other fruit and vegetable products included), outcomes (method of assessment, psychological measure), and findings (observations, conclusions).

In order to assess bias and the general quality of the studies, based on the Cochrane recommendations for the tools for assessing the methodological quality or the risk of bias in non-randomized studies [31], the Newcastle–Ottawa Scale (NOS) [32] was applied, which is used commonly [33]. Each included study was assessed for the following criteria: For case-control studies—selection (scale from 0 to 4), comparability (scale from 0 to 2), and exposure (scale from 0 to 3); and for cohort studies—selection (scale from 0 to 4), comparability (scale from 0 to 2), and outcome (scale from 0 to 3). The results were interpreted based on the commonly assumed criteria and attributed to the following categories: very high risk of bias (0–3 NOS points), high risk of bias (4–6 NOS points), and low risk of bias (7–9 NOS points) [34].

The following types of outcomes were included to the presented systematic review: General and mental well-being, quality of life, sleep quality, life satisfaction, and mood (general outcomes); flourishing, self-efficacy, curiosity, creativity, optimism, self-esteem, and happiness (positive outcomes); stress, nervousness, anxiety, minor psychiatric disorders, distress, depressive symptoms, depression, and attempted suicide (negative outcomes). Due to the fact that numerous various outcomes were included, while the number of studies for each outcome differed from only one study, to a lot of studies (as for depression or depressive symptoms), it was not possible to summarize the results in the form of meta-analysis, as it requires including comparable studies only (for one type of outcome). As not only outcomes, but also way to express risk factor (namely fruit and/or vegetable intake), studied populations, and settings were not comparable, the studies may not be treated as sufficiently similar to be able to reanalyze the data in the form of meta-analysis, but in the future, while the number of such studies will be higher, for each outcome, an adequate meta-analysis will be valuable to conclude. Taking it into account, based on the data extracted and the assessment of the general quality of the study, a narrative synthesis of the findings from the included studies was performed, which was structured around the type of outcome. 

## 3. Results

The list of studies included to the systematic review is presented in Supplementary Table S2. The basic study details and design of observation for the studies included to the systematic review is presented in Table 1. The studies presented in all the tables are listed accordingly based on the year of publication. Among 61 included studies, the majority were conducted for European countries (20 studies), Asian countries (14 studies), or United States of America (USA) (11 studies), but also those conducted for African (1 study), or South American country (1 study), Australia or New Zealand (7 studies), or Canada (3 studies) were included and some of them were conducted for mixed countries (4 studies). The studied populations were mainly adults (including young, middle aged, and older ones, or only some of indicated groups) (37 studies), as well as only young ones (12 studies), middle aged ones (3 studies), or old ones (9 studies). It should be indicated that 3 included studies were conducted in a specific populations, while the health-related inclusion criteria for the study were defined as prediabetes and/or prehypertension [35], being after coronary artery bypass grafting surgery [36], or having excessive body mass [37].

The characteristics of the study participants for the studies included to the systematic review is presented in Supplementary Table S3. The characteristics of the study exposure and outcomes for the studies included to the systematic review is presented in the Table 2. Among 61 included studies, for the majority of them, a food frequency questionnaire, or a rapid screener was applied (32 studies), or a specific questionnaire to assess the diet quality (1 study), or a simple question about fruit and vegetable intake (22 studies), but for 4 studies, the method of dietary recall or record was applied, either alone or combined with previously indicated methods, and for 2 studies the applied method was not specified. To assess the mental health, various aspects of it were analyzed in the studies, while either a single aspect or some aspects combined were assessed, including mainly depressive symptoms (18 studies), depression (15 studies), general and mental well-being (9 studies), stress (8 studies), distress (8 studies), quality of life (7 studies), mood (5 studies), or anxiety (5 studies), but also in some of them happiness (4 studies), life satisfaction (3 studies), or optimism (2 studies) were analyzed, and in single studies only: sleep quality, flourishing, self-efficacy, curiosity, creativity, self-esteem, nervousness, minor psychiatric disorders, or attempted suicide were indicated.

The characteristics of the study findings for the studies included to the systematic review accompanied by the quality assessment based on the total score for the Newcastle–Ottawa Scale is presented in the Table 3. The detailed results of the quality assessment based on the total score for the Newcastle–Ottawa Scale for categories of selection, comparability and exposure/outcome are presented in Supplementary Table S4. Among 61 included studies, for the majority of them, a statistically significant influence of fruit and/or vegetable consumption on mental health was proven, but there were also some studies, for which it was proven only in case of some factors associated with mental health and not for all assessed ones and only few studies, for which it was not proven that there is any association [37,38,39]. While concluding, authors of the included studies emphasized the existing association, but also among most important remarks, they indicated risks resulting from not following a recommendation to consume at least 5 portions of fruit and vegetable each day (2 studies), or they indicated especially beneficial groups of fruit and vegetables (2 studies).

## 4. Discussion

The systematic review of the observational studies analyzing the association between the intake of fruit and vegetable and mental health in adults revealed the possible beneficial influence of the indicated products. This association was studied for various aspects of mental health, ranging from general and mental well-being [35,45,49,62,68,77,82,84,94], quality of life [36,39,42,43,47,55,70], sleep quality [55], life satisfaction [50,66,82], flourishing [50], mood [50,62,68,77,81], self-efficacy [56], curiosity [68], creativity [68], optimism [91,93], self-esteem [90], stress [40,48,67,71,73,80,89,92], nervousness [82], or happiness [52,59,65,82], to anxiety [35,48,50,54,67], minor psychiatric disorders [46], distress [38,46,54,58,69,72,77,82], depressive symptoms [37,41,43,50,51,53,59,60,63,64,73,74,76,79,85,86,87,89], depression [35,42,44,48,54,57,61,67,69,75,77,78,83,84,92], or attempted suicide [88]. 

Moreover, the indicated effect was studied and stated not only for fresh fruits and vegetables [35,36,37,38,39,40,41,42,43,44,45,46,47,48,49,50,51,52,53,54,55,56,57,58,59,60,61,62,63,64,65,66,67,68,69,70,71,72,73,74,75,76,77,78,79,80,81,82,83,84,85,86,87,88,89,90,91,92,93,94,95] but also for fruit and vegetable products, such as juices [37,39,40,48,49,51,52,54,56,60,61,65,70,72,82,83,88,92,94], dried [37,49,54,66], and canned fruits and vegetables [37,38,49,50,58,65,66], salads [39,40,44,49,52,54,58,61,62,72,73,74,89,93,94], soups [39,40,62], or ketchup [39,79], and even for potatoes in some studies that included it to the total intake of fruits and vegetables [39,74]. This corresponds to the general conclusions of some studies that not only the intake of fruit and vegetable should be increased, but also at least 5 portions of fruits or vegetables must be taken daily as recommended to observe a positive influence on the general mental health [57,72].

The reason behind promoting the consumption of 5 portions of fruits or vegetables daily is that the Food and Agriculture Organization of the United Nations (FAO) and WHO have recommended to consume a minimum of 400 g of fruits and vegetables per day, excluding potatoes and other starchy tubers, with an estimated serving size of 80 g [96]. This recommendation is also supported by a number of prominent experts, boards, and associations, such as the National Health Service (NHS) in Great Britain [97], American Heart Association (AHA) [98], Centers for Disease Control and Prevention (CDC) [99], and Office of Disease Prevention and Health Promotion (ODPHP) in the United States of America [100]. Moreover, not only the role of raw fruits and vegetables but also that of the processed fruits and vegetables, including frozen, canned, or cooked ones [101], as well as juices [102], is emphasized to meet the recommended intake.

Increasing the intake of fruits and vegetables to the recommended level may result in a noticeable and measurable effect, as was stated in some included studies. An increase in the consumption of fruits and vegetables by one portion a day leads to a 0.133-unit improvement in the mental well-being assessed by GHQ-12 scale [45], while the consumption of 7–8 servings a day leads to meaningful changes in positive affect [81] and consumption of 8 portions a day leads to a 0.24-unit increase in life satisfaction (equivalent to the psychological gain of moving from unemployed status to employed) [66]. However in general, any increase in the consumption of fruits and vegetables results in the improvement of well-being, enhances happiness, and decreases depressive symptoms, with the strongest effect observed for 6 servings a day [59], 7 servings a day [82], or more than 8 servings a day (combined with breakfast every day and 3 meals in addition to 1–2 snacks per day) [65], depending on the studied group.

It must be indicated that among the included studies, some highlighted not only the general effect of fruits and vegetables but also the influence of specific types, such as citrus [48], berries [93], green leafy vegetables [48], green salad [70], and tomatoes [79]. Simultaneously, among the raw fruits and vegetables, the following were indicated as specially related to better mental health: Bananas, apples, citrus, berries, grapefruit, kiwifruit, carrots, lettuce, cucumber, and green leafy vegetables, particularly spinach [50]. Authors of the indicated studies have no definite explanation why those fruits or vegetables may be especially valuable, similarly as the general mechanism of the influence on mental health is still unknown [18]. However, it may be supposed that the positive influence should be attributed to a specific nutritional value, as a combination of high content of compounds positive for mental health and, at the same time, low content of those negative for mental health, as indicated by LaChance and Ramsey [12]. However, such assumption does not take into account the potential interactions between nutrients in food product and between food products in the diet, so it must be also emphasized that in fact estimating the independent effect of only one type of food products (fruits and vegetables), or nutrients may be hard to conduct, due to other food products, or nutrients, that are interfering.

While presenting the results of the included studies analyzing the association between the intake of fruit and vegetable and mental health in adults, it must be emphasized that the risk of bias varied from very high to low (defined based on the commonly assumed criteria [34]) as shown by the total NOS score. Taking this into account, the highest attention must be paid to those studies interpreted as having a low risk of bias and the highest quality. Within such studies, it was observed that high total intake of fruits and vegetables and some of their specific subgroups, including berries, might be associated with a high level of optimism [93], while this association is independent of interfering factors [91]. High intake of fruits and vegetables was also associated with a higher level of self-efficacy [56], as well as a low level of psychological distress [58], ambiguity, and cancer fatalism [56]. In addition, it was observed that high total intake of fruits and vegetables and some of their specific subgroups, including citrus and green leafy vegetables, might be associated with a lower risk of depression [48], but other health-related factors may also play a role in this association [69,85]. Furthermore, it was highlighted that increasing physical activity may be necessary to benefit from the positive effect of the consumption of fruit and vegetable to protect against depressive symptoms [64].

In spite of the fact that the vast majority of the included studies presented compatible conclusions that mental health benefits can be attained with increased consumption of fruits and vegetables, some limitations of this systematic review must be indicated. The main issue results from the fact, that association between fruit or vegetables intake and mental health does not allow to state unambiguously that the intake influences mental health, as the reverse causation is also possible, so only based on experimental study it may be concluded with no doubts, that such influence exists. Moreover, it must be emphasized that no uniform definition of fruit and vegetables was established in analyzed studies as various fruit and vegetable products were either included or excluded from this group, that may also have influenced the observed association. Last but not least, the included studies were conducted using various methodologies, in different populations, and involved diverse measures of intake of fruits and vegetables. Therefore, further studies should be conducted covering all the aspects of mental health in various populations and using the similar methodology, to analyze the association between the consumption of fruits and vegetables and mental health in detail. 

## 5. Conclusions

The vast majority of the included studies indicated that the intake of fruits and/or vegetables and their specific subgroups, as well as processed fruits and vegetables, seems to have a positive influence on mental health. Therefore, the general recommendation to consume at least 5 portions of fruit and vegetable a day may be beneficial also for mental health.

## Figures and Tables

**Table 1:** General details of the study and design for the studies included to the systematic review.

Ref.	Study Details			Observation	
	Author (Year)	Study Design	Country/Location	Study Group	Time
[40]	Chang et al., 2019	Cross-sectional study within Special Supplemental Nutrition Program for Women, Infants, and Children in Michigan, USA	United States of America (USA)	Non-Hispanic women	May to August 2010
[41]	Cheng et al., 2019	Observational study	China/Linyi, Shandong Province	Middle-aged Chinese population	May 2016 to June 2017
[42]	Gehlich et al., 2019	Longitudinal population-based study within Survey of Health, Ageing and Retirement in Europe (SHARE)	Austria, Belgium, Denmark, France, Germany, Italy, Netherlands, Slovenia, Spain, Sweden, Switzerland	Older adults	2011 and 2013 waves of the study
[43]	Gehlich et al., 2019	Cross-sectional, population-based study based on the WHO Study on Global Ageing and Adult Health (SAGE)	China, India, Mexico, Russia, South Africa, Ghana	Adults ≥ 50 years	2007–2010
[44]	Goh et al., 2019	Cross-sectional, population-based study within Well-being of the Singapore Elderly (WiSE) Study	Singapore	Adults ≥ 60 years	December 2013
[45]	Ocean et al., 2019	Longitudinal study within the UK Household Longitudinal Survey (UKHLS)	United Kingdom (UK)	General population	2010–2017 waves of the study
[35]	Pengpid et al., 2019	Longitudinal study within a lifestyle intervention trial	Thailand/Nakhon Pathom province	Temple members with prediabetes and/or prehypertension	2016–2018
[46]	Salvatore et al., 2019	Cross-sectional study—10,001 Dalmatians Study	Croatia/Split and Island of Korčula	Adults	2007–2015
[47]	Azupogo et al., 2018	Cross-sectional study	Ghana/Tolon and Savelugu Districts	Rural women in fertile age	April to May 2016
[48]	Baharzadeh et al., 2018	Cross-sectional study	Iran/Khorramabad	Women attending health centers	May to October 2017
[49]	Boehm et al., 2018	Observational population-based study within English Longitudinal Study of Ageing (ELSA)	UK	Adults ≥ 50 years	2006–2013 waves of the study
[50]	Brookie et al., 2018	Cross-sectional study	New Zealand, USA	Young adults aged 18–25 recruited as part of psychology course at university, or through an online crowdsourcing marketplace	March to June 2017
[51]	Hoare et al., 2018	Longitudinal, population-based study	USA	Adolescents at baseline, adults in follow-up	1994–1995 and 2007–2008 waves of the study
[52]	Jyväkorpi et al., 2018	Cross-sectional study in longitudinal Helsinki Businessmen Study (HBS) cohort	Finland/Helsinki	Oldest-old, home-dwelling men	2016
[53]	Pagliai et al., 2018	Cross-sectional study—Mugello Study	Italy/Florence	Nonagenarians (90–99 years)	Not specified
[54]	Saghafian et al., 2018	Cross-sectional study within Study on the Epidemiology of Psychological, Alimentary Health and Nutrition (SEPAHAN)	Iran	Adults working in health centers	2010 ^1^
[55]	Tan et al., 2018	Cross-sectional study	Germany, Netherlands	Adults ≥ 20 years	2013–2015
[56]	Welch and-- Ellis 2018	Cross-sectional, population-based study—Health Information National Trends Survey (HINTS)	USA	Adults	2011–2017 waves of the study
[57]	Bishwajit et al., 2017	Cross-sectional study based on World Health Survey of WHO	Bangladesh, India, Nepal	Adults	2002–2004
[58]	Nguyen et al., 2017	Longitudinal, cross-sectional, population-based study—Sax Institute’s 45 and Up Study	Australia/New South Wales	Adults	2006–2008, 2010
[59]	Peltzer and Pengpid 2017	Cross-sectional study	Bangladesh, Barbados, Cameroon, China, Colombia, Egypt, Grenada, India, Indonesia, Ivory Coast, Jamaica, Kyrgyzstan, Laos, Madagascar, Malaysia, Mauritius, Namibia, Nigeria, Pakistan, Philippines, Russia, Singapore, South Africa, Thailand, Tunisia, Turkey, Venezuela, Vietnam	University students	Not specified
[60]	Ribeiro et al., 2017	Cross-sectional and longitudinal study—African American Health (AAH)	USA/Missouri, St. Louis	Urban-dwelling African Americans	2007–2010
[61]	Richard et al., 2017	Cross-sectional, population-based study—based on COhorte LAUSannoise(CoLaus)/Psychiatric CoLaus (PsyCoLaus) and Swiss Health Survey (SHS)	Switzerland	Adults ≥ 40 years	2009–2012
[62]	Warner et al., 2017	Cross-sectional study	USA/New England	University students	2013–2014
[63]	Wolniczak et al., 2017	Observational, population-based study within Health Questionnaire of the Demographic Health Survey—Encuesta Demográfica y de Salud Familiar (ENDES)	Peru	Adults	2014
[64]	Chi et al., 2016	Longitudinal study—Taiwan Longitudinal Survey on Aging (TLSA)	Taiwan	Adults ≥ 53 year	1999, 2003
[65]	Lesani et al., 2016	Cross-sectional study	Iran	Students of Qazvin University of Medical Sciences	Not specified
[66]	Mujcic and Oswald 2016	Longitudinal, population-based study—Household, Income, and Labour Dynamics in Australia (HILDA) Survey	Australia	Adolescents or adults at baseline, adults in follow-up	2007, 2009, 2013
[67]	Beezhold et al., 2015	Observational study	USA, Canada and other countries (16%)	Adults	2013
[68]	Conner et al., 2015	Micro-longitudinal study within Daily Life Study	New Zealand	Students at the University of Otago	2013
[69]	Kingsbury et al., 2015	Longitudinal population-based study—National Population Health Survey (NPHS)	Canada	Adults	2002–2011
[70]	Kwon et al., 2015	Observational study within Racial and Ethnic Approaches to Health Across the United States (REACH US)	USA/New York	Minority groups in ethnic enclaves	2009–2012
[71]	Papier et al., 2015	Cross-sectional study	Australia	First year undergraduate students of Griffith University	2012–2013
[72]	Richard et al., 2015	Cross-sectional, population-based study—2012 Swiss Health Survey	Switzerland	Adolescents and adults ≥ 15 years	2012–2013
[73]	El Ansari et al., 2014	Cross-sectional study	England, Wales, Northern Ireland	Undergraduate students	2007–2008
[74]	Mihrshahi et al., 2014	Longitudinal study	Australia	Women	2004, 2007, 2010
[75]	Rutledge et al., 2014	Cross-sectional study—Women’s Ischemia Syndrome Evaluation (WISE)	USA	Women	1996–2000 with median of 5.9 years of follow up
[37]	Whitaker et al., 2014	Cross-sectional study—Sisters Taking Action for Real Success (STARS)	USA/Columbia	Overweight and obese women from economically disadvantaged neighborhoods	June to July 2008 ^1^
[76]	Akbaraly et al., 2013	Cross-sectional study—Whitehall II	UK	Adults	1985–1988, 1991–1993, 2003–2004, 2008–2009
[38]	Bhattacharyya et al., 2013	Cross-sectional study	India/Goa	Adults	April 2004 to January 2005
[77]	McMartin et al., 2013	Cross-sectional, population-based study within Canadian Community Health Survey (CCHS)	Canada	Adolescents and adults ≥ 12 years	2000–2009
[78]	Meyer et al., 2013	Cross-sectional, population-based study—Australian National Nutrition Survey (NNS), Australian National Health Survey (NHS)	Australia	Adults	1995
[79]	Niu et al., 2013	Cross-sectional, population-based study—Tsurugaya Project	Japan/Sendai	Adults ≥ 70 year	2002
[80]	Roohafza et al., 2013	Cross-sectional study within Isfahan Healthy Heart Program (IHHP)	Iran/Isfahan, Arak, Najafabad	Adults	Not specified
[81]	White et al., 2013	Micro-longitudinal study	New Zealand/Otago	Undergraduate students	April 2008 to August 2009
[82]	Blanchflower et al., 2012	Cross-sectional, population-based study of the data from Welsh Health Survey of 2007–2010, Scottish Health Survey of 2008 and Health Survey of England in 2008	UK	Adults	2007–2010
[83]	Davison and Kaplan 2012	Cross-sectional study	Canada	Adult members of the Mood Disorders Association of British Columbia (MDABC)	Not specified
[84]	Payne et al., 2012	Case-control study within longitudinal clinical study NeuroCognitive Outcomes of Depression in the Elderly (NCODE)	USA	Adults ≥ 60 year	1999–2007
[85]	Tsai et al., 2011	Prospective population-based study—Survey of Health and Living Status of the Elderly in Taiwan (SHLSET)	Taiwan	Adults ≥ 65 years	1999, 2003
[36]	Tung et al., 2011	Cross-sectional study	Taiwan/Taipei	Adults after coronary artery bypass grafting surgery	February to June 2009
[39]	Chai et al., 2010	Longitudinal study	USA/Hawaii	Adults	2-year study period
[86]	Konttinen et al., 2010	Cross-sectional, population-based study—National Cardiovascular Risk Factor Survey (The FINRISK Study)/Dietary, Lifestyle and Genetic Determinants of Obesity and Metabolic Syndrome (DILGOM substudy)	Finland	Adults	2007
[87]	Mamplekou et al., 2010	Longitudinal population-based study—Mediterranean Islands Elderly Study (MEDIS)	Cyprus, Greece	Adults ≥ 65 years	2005–2007
[88]	Li et al., 2009	Longitudinal cross-sectional study—Third National Health and Nutrition Examination Survey (NHANES III)	USA	Adults	1988–1994
[89]	Mikolajczyk et al., 2009	Cross-sectional study—Cross National Student Health Survey (CNSHS)	Germany, Poland, Bulgaria	First-year students	2005
[90]	Elfhag and Rasmussen 2008	Cross-sectional study in Parental Influences on Their Children’s Health (PITCH) data set	Sweden	Women	2000
[91]	Giltay et al., 2007	Longitudinal study within Zutphen Elderly Study	Netherlands	Elderly community-living men	1985, 1990, 1995, 2000
[92]	Liu et al., 2007	Cross-sectional study—China Seven City Study	China	College students	November 2003 to January 2004
[93]	Kelloniemi et al., 2005	Cross-sectional, population-based study	Finland	31-year-old adults	1997–1998
[94]	Sarlio-Lähteenkorva et al., 2004	Cross-sectional study within Helsinki Health Study	Finland/Helsinki	Adults 40–60 years	2000–2001
[95]	Cook and Benton 1993	Cross-sectional, population-based study	Wales/Swansea	Adults	Not specified

^1^ Data provided by authors on request.

**Table 2:** Characteristics of the study exposure and outcomes for the studies included to the systematic review.

Ref.	Exposure			Outcomes	
	Assessment	Measure of Fruit and Vegetable	Other Fruit/Vegetable Products	Assessment	Psychological Measure
[40]	Rapid Food Screener	Frequency of consumption	Fruits including juices Vegetables including salads, soups	Stress	Perceived Stress Scale
[41]	Semi-quantitative food frequency questionnaire	Portion size, frequency of consumption	Not specified	Depressive symptoms	Center for Epidemiological Studies Depression Scale (CESD-10)
[42]	Question about fruit and vegetable consumption	Frequency of consumption	Not specified	(1) Depression (2) Quality of life	(1) EURO-D scale (depression scale) (2) CASP-12 with subscales for Control, Autonomy, Self-realization and Pleasure
[43]	Question about fruit and vegetable consumption on a typical day with a list of country-specific examples of fruits and vegetables	Frequency of consumption	Not specified	(1) Depressive symptoms (2) Quality of life	(1) Number of depressive symptoms during the previous 2 weeks (loss of appetite, slowed-down thinking, problems falling asleep, waking up too early, difficulties concentrating, slowing down in moving around, feeling anxious or worried, being restless, feeling negative about oneself, feeling hopeless, losing interest in sex, suicidal ideation, suicidal behavior) (2) 8-items version of the WHO Quality of Life scale (WHOQOL)
[44]	Question about fruit and vegetable consumption over the last 3 days	Frequency of consumption	Vegetables including salads	Depression and subsyndromal depression	Geriatric Mental State (GMS)
[45]	Questions about fruit and vegetable consumption on a typical day when they are consumed, fruit and vegetable consumption on a typical week	Portion size, frequency of consumption	Not specified	Mental well-being	12-item General Health Questionnaire (GHQ-12)
[35]	WHO STEPS Instrument	Frequency of consumption	Not specified	(1) Mental components of health (2) Major depression (3) Anxiety	(1) Short Form (SF-8) Health Survey (2) Patient Health Questionnaire-9 (PHQ-9) (3) Generalized Anxiety Disorder (GAD-7)
[46]	Food frequency questionnaire with 55 food items	Frequency of consumption recalculated into Mediterranean Diet Serving Score (MDSS)	Not specified	Minor psychiatric disorders and psychological distress	General Health Questionnaire-30 (GHQ-30)
[47]	Semi-quantitative food frequency questionnaire with 27 food items	Frequency of consumption	Not specified	Health-related quality of life (HR-QoL)	Short Form 36-Health Survey (SF-36), including Mental Health Component (MH)
[48]	Semi-quantitative food frequency questionnaire with 147 food items and 14 items related to local spices and vegetables	Frequency of consumption converted to g/day	Fruits including juices	Depression, anxiety and stress	The Depression, Anxiety, Stress Scales (DASS, 21-items)
[49]	Questions about fruit and vegetable consumption on a previous day	Frequency of consumption	Fruits including canned, dried, fruit juices Vegetables including salads	Psychological well-being	17-items from the Control, Autonomy, Satisfaction, Pleasure Scale (CASP-17)
[50]	Questions about number of days a week when fruit and vegetables are consumed, and consumption on a typical day when they are consumed	Frequency of consumption for raw and processed ones	Fruit including canned Vegetables including canned	(1) Depressive symptoms (2) Anxiety (3) Negative and positive mood (4) Life satisfaction (5) Flourishing	(1) Centre for Epidemiological Depression Scale (CESD) (2) 7-items Hospital Anxiety and Depression Scale—Anxiety Subscale (HADS-A) (3) Question about 12 negative (hostile, stressed, irritable, angry, anxious, annoyed, nervous, tense, hopeless, unhappy, dejected, sad) and 12 positive affects (enthusiastic, excited, energetic, joyful, happy, cheerful, pleasant, good, relaxed, calm, content, satisfied) (4) Satisfaction with Life Scale (SWLS) (5) Flourishing Scale
[51]	Question about fruit and vegetable consumption on a previous day	Frequency of consumption	Fruits including fruit juices Vegetables including vegetable juices	Depressive symptoms	Center for Epidemiological Studies Depression Scale (CESD-10)
[52]	Nutritional questionnaire to assess intake of products for Mediterranean Diet adherence score (MeDi) and Index of Diet Quality (IDQ)	Frequency of consumption	100% juice as a separate group Vegetables including salads	Perceived happiness	Visual Analog Scale of Happiness (0–100 mm)
[53]	Not specified	Frequency of consumption recalculated into Mediterranean Diet Score	Not specified	Depressive symptoms	Geriatric Depression Scale (GDS)
[54]	Semi-quantitative food frequency questionnaire with 106 food items	Total fruit and total vegetables intake	Fruit including juices, dried and herbs Vegetables including salads	(1) Anxiety and depression (2) Psychological distress	(1) Iranian-validated version of Hospital Anxiety and Depression Scale (HADS) (2) Iranian-validated version of General Health Questionnaire (GHQ)
[55]	Question about eating 5 portions of fruit and vegetables daily during last weeks	5-point Likert scale for following recommendation	Not specified	(1) Restful sleep; (2) Sleep quality; (3) Quality of life and subjective health	(1) Center for Epidemiological Studies Depression Scale (CESD-10) item—level of restful sleep; (2) World Health Organization Quality of Life (WHOQOL-BREF) Questionnaire question—level of satisfaction with sleep; (3) WHOQOL-BREF Questionnaire question—level of quality of life
[56]	Two items from the National Cancer Institute Quick Food Scan	Frequency of consumption	Fruit including 100% pure juices Vegetables including 100% pure juices	Health-Related Self-Efficacy	Question: “Overall, how confident are you about your ability to take good care of your health?” using a 5-point Likert scale ranging from (1) completely confident to (5) not at all confident
[57]	Question about fruit consumption on a typical day	Frequency of consumption	Not specified	Self-reported depression status	(1) question: “During the last 12 months, have you had a period lasting several days when you felt sad, empty or depressed?” Yes/No (2) question: “Overall in the last 30 days, how much of a problem did you have with feeling sad, low or depressed?” None/Mild/Moderate/Severe/Extreme
[58]	Validated questions about fruit and vegetable consumption on a typical day	Frequency of consumption	Fruits including canned Vegetables including salads	Psychological distress	10-items Kessler Psychological Distress Scale (K10)
[59]	Question about fruit and vegetable consumption on a typical day	Frequency of consumption	Not specified	(1) Happiness (2) Depressive symptoms	(1) Happiness Scale (2) Center for Epidemiological Studies Depression Scale (CESD-10)
[60]	Question about fruit and vegetable consumption on a typical day	Frequency of consumption, intake	Fruit including juices	Clinically-relevant levels of depressive symptoms	Center for Epidemiological Studies Depression Scale (CES-D)
[61]	Either semi-quantitative food frequency questionnaire with 90 food items (CoLaus/PsyCoLauS), or question about fruit and vegetable consumption on a typical day (SHS)	Frequency of consumption	Fruits including juices Vegetables including salads and juices	Depression	Either semi-structured Diagnostic Interview for Genetic Studies (DIGS) (CoLaus/PsyCoLauS), or Patient Health Questionnaire (PHQ-9) (SHS)
[62]	The National Cancer Institute (NCI) All Day Fruit and Vegetable Screener	Portion size, frequency of consumption	Vegetables including salads and vegetable soups	(1) Well-being (2) Positive affect and negative affect	(1) Satisfaction with Life Scale (LS) (2) The Positive and Negative Affect Schedule (PANAS)
[63]	WHO STEPS Instrument	Number of portions	Not specified	Depressive symptoms (including major depressive syndrome)	Patient Health Questionnaire (PHQ-9)
[64]	Validated semi-quantitative questionnaire	Frequency of consumption	Not specified	Depressive symptoms	Center for Epidemiological Studies Depression Scale (CESD-10)
[65]	Questions about number of days a week when fruit and vegetables are consumed, and consumption on a typical day when they are consumed	Frequency of consumption	Fruit including canned and juices Vegetables including canned and juices	Happiness score	Oxford Happiness Questionnaire (OHQ)
[66]	Questions about number of days a week when fruit and vegetables are consumed, and consumption on a typical day when they are consumed	Frequency of consumption	Fruit including canned and dried Vegetables including canned	Self-reported life satisfaction	(1) Question about subjective assessment how satisfied with his life is respondent (2) Medical Outcomes Short Form (SF-36)—question about being a happy person
[67]	Question about fruit and vegetable included to diet at least monthly; Question about fruit and vegetable consumption on a typical day	Frequency of consumption	Not specified	Depression, anxiety and stress	The Depression, Anxiety, Stress Scales (DASS, 21-items)
[68]	Question about fruit and vegetable consumption on a present day	Frequency of consumption	Juices, dried fruits and vegetables excluded	(1) Daily eudaemonic well-being (2) Daily curiosity (3) Daily creativity (4) Daily affect	(1) Adaptation of the Flourishing Scale (8-items) (2) Curiosity and Exploration Inventory (7-items) (3) Question about subjective assessment of own creativity level on a present day (4) Question about subjective assessment of own affects on a present day—9 items for positive affects (calm, content, relaxed, happy, cheerful, pleasant, energetic, enthusiastic, and excited) and 9 for negative affcts (sad, dejected, depressed, nervous, anxious, tense, angry, irritable, and hostile
[69]	Question about fruit and vegetable consumption on a typical day	Frequency of consumption	Juices excluded	(1) Major depression (2) Psychological distress	(1) Composite International Diagnostic Interview Short Form (CIDI-SF) (2) Kessler Psychological Distress Scale (K6)
[70]	6-items food frequency screener	Frequency of consumption	Fruit juices and green salad included in the screener as a separate question	Quality of life	Health-related quality of life (HRQOL) (including self-reported mental health, days of poor mental health in the past month, days of limited activities because of poor physical/mental health in the past month)
[71]	Commonwealth Scientific and Industrial Research Organization food frequency questionnaire (CSIRO FFQ)	Frequency of consumption	Fruit/vegetables excluding juices	Stress	Depression Anxiety Stress Scale (DASS)—Stress subscale
[72]	Food frequency questionnaire including questions about number of days a week when fruit and vegetables are consumed, and consumption on a typical day when they are consumed	Frequency of consumption, following recommendations	Fruits including juices Vegetables including salads and juices	Psychological distress	5-items Mental Health Inventory (MHI-5)
[73]	Food frequency questionnaire	Frequency of consumption	Vegetables including salads	(1) Stress (2) Depressive symptoms	(1) Cohen’s Perceived Stress Scale (PSS-4 items) (2) Modification of the Beck Depression Inventory (MBDI)
[74]	Question about fruit and vegetable consumption on a typical day	Frequency of consumption	Vegetables including salads and potatoes	Depressive symptoms	Center for Epidemiological Studies Depression Scale (CESD-10)
[75]	1998 Block Food Frequency Questionnaire for Adults	Frequency of consumption	Not specified	Depression	The Beck Depression Inventory (21-items); Current use of antidepressants; Self-reported history of treatment for depression
[37]	Three 24-h dietary recalls	Frequency of consumption	Fruits including canned, dried, juices Vegetables including juices	Depressive symptoms	Center for Epidemiological Studies Depression Scale (CESD-10)
[76]	Semi-quantitative food-frequency questionnaire with 127 food items	Intake recalculated into the Alternative Healthy Eating Index (AHEI), including among others component of fruits and of vegetables	Not specified	Depressive symptoms	Center for Epidemiological Studies Depression Scale (CESD-10); Self-reported use of antidepressants
[38]	Food frequency questionnaire with 63 food items	Frequency of consumption	Fruit including canned	Psychological distress	Kessler Psychological Distress Scale (K10)
[77]	Question about fruit and vegetable consumption on a typical day	Frequency of consumption	Juices excluded	(1) Major depressive episode (2) Psychological distress (3) Self-perceived mental health (4) Physician-diagnosed mood and anxiety disorder	(1) Composite International Diagnostic Interview-Short Form (CIDI-SF) (2) Kessler Psychological Distress Scale (K6) (3) Question about self-perceived mental health (4) Question about chronic conditions diagnosed by a health professional for a mood disorder, such as (A) depression, bipolar disorder, mania or dysthymia, (B) anxiety disorder such as a phobia, obsessive–compulsive disorder or a panic disorder
[78]	24-h dietary recall; Question about frequency of consumption	Number of portions, frequency of consumption	Not specified	Depression	Question about any recent illness (medical conditions during previous 2 weeks) or long-term illness (lasted at least 6 months), screened for depression, based on ICD-10
[79]	Brief self-administered Diet History Questionnaire (BDHQ) with 75 food items	Frequency of consumption	Tomato products including tomato ketchup, stewed tomato, or tomato stew	Depressive symptoms	30-items Japanese version of Geriatric Depression Scale (GDS)
[80]	Food frequency questionnaire with 49 food items	Frequency of consumption	Not specified	Psychologic stress	General Health Questionnaire-12 (GHQ-12)
[81]	Question about fruit and vegetable consumption on a typical day	Frequency of consumption	Juices and dried fruits excluded	Negative and positive affects	Questions about 9 negative (depressed, sad, unhappy, anxious, nervous, tense, angry, hostile, short-tempered) and 9 positive affects (calm, content, relaxed, cheerful, happy, pleased, energetic, enthusiastic, excited)
[82]	Not specified	Frequency of consumption	Fruit including orange juice	Depending on the study: (1) Life satisfaction (2) Well-being (3) Mental ill-being/mental distress (4) Happiness (5) Nervousness (6) Being downhearted and low	Depending on the study: (1) Question about self-reported life satisfaction (2) The Warwick-Edinburgh Mental Wellbeing Scale (WEMWBS) (3) General Health Questionnaire GHQ (including mental ill-being/mental distress questions) (4) Question about self-reported being happy (5) Question about self-reported being nervous (6) Question about self-reported being downhearted and low
[83]	3-day dietary record; Validated food frequency questionnaire	Frequency of consumption	Fruits including juices and nectars	Depression	Structured Clinical Interview for DSM-IV Axis I Disorders, Global Assessment of Functioning (GAF) Scale, Hamilton Depression Scale (Ham-D), Young Mania Rating Scale (YMRS)
[84]	1998 Block Food Frequency Questionnaire	Frequency of consumption	Not specified	Depression; Self-reported physical health	Duke Depression Evaluation Schedule (sections of the National Institute of Mental Health (NIMH) Diagnostic Interview Schedule which assesses depression, as well as items on self-reported physical health)
[85]	Question about fruit and vegetable consumption on a typical week	Frequency of consumption	Not specified	Depressive symptoms (interpreted as having a risk of depression)	Center for Epidemiological Studies Depression Scale (CESD-10)
[36]	Chinese Food Frequency Questionnaire-Short Form (Short C-FFQ)	Frequency of consumption	Not specified	Quality of life	Taiwanese version of the Short Form 36-Health Survey (SF-36)
[39]	NCI Fruit and Vegetable screener	Frequency of consumption of 9 categories of fruits and vegetables	Fruit and vegetables (including fruit, fruit juices, salad, beans, French fries, other potatoes, tomato sauce, vegetable soups and other vegetables)	Quality of life	SF-12 Health Survey
[86]	132-item food frequency questionnaire	Frequency of consumption	Mayonnaise salads excluded from vegetables	Depressive symptoms	Center for Epidemiological Studies Depression Scale (CESD-10)
[87]	Semi-quantitative food-frequency questionnaire	Frequency of consumption	Not specified	Depressive symptoms	Shortened version of Geriatric Depression Scale (GDS)
[88]	Qualitative 60-items food frequency questionnaire; 24-h dietary recall	Frequency of consumption	Fruits including juices	Attempted suicide	Simple question (“Have you ever attempted suicide?”), according to the standardized criteria established in the Diagnostic Interview Schedule
[89]	Food frequency questionnaire	Frequency of consumption	Vegetables including salads	(1) Perceived stress (2) Depressive symptoms	(1) Cohen’s Perceived Stress Scale (PSS—14 items) (2) Modification of Beck Depression Inventory (M-BDI)
[90]	Food frequency questionnaire	Frequency of consumption	Not specified	Self-esteem	Harter Self-Perception Profile for Adults (SPPA)
[91]	Cross-check dietary history method	Frequency of consumption, intake	Not specified	Dispositional optimism	4-item questionnaire—“I still expect much from life,” “I do not look forward to what lies ahead for me in the years to come,” “My days seem to be passing by slowly,” and “I am still full of plans”
[92]	Food frequency questionnaire	Frequency of consumption	Fruits including juices	(1) Stress (2) Depression	(1) Perceived Stress Scale (PSS) (2) Center for Epidemiological Studies Depression Scale (CES-D)
[93]	Food frequency questionnaire with 32 food items	Frequency of consumption	Vegetables including salads	Dispositional optimism	Life Orientation Test (LOT-R)
[94]	Food frequency inventory	Frequency of consumption	Fruits including juices Vegetables including salads	Mental health status	(1) General Health Questionnaire (GHQ-12) (2) History of lifetime mental diagnosis (for depression, anxiety, eating disorder, any other mental health problem)
[95]	Food frequency questionnaire	Frequency of consumption	Fruits including dried, canned, pure juices Vegetables including salads, potatoes (not chips)	Anxiety, depression	General Health Questionnaire-30 (GHQ-30)

**Table 3:** Characteristics of the study findings for the studies included to the systematic review accompanied by the quality assessment based on the total score for the Newcastle–Ottawa Scale.

Ref.	Findings		Quality *
	Observation	Conclusion	
[40]	Women with high stress consumed significantly less fruits (*p* = 0.01) and vegetables (*p* = 0.02) than women with low stress, with effect sizes of d = 0.24 and 0.25, respectively, for the between-group differences.	Nutrition counselling on increasing fruit and vegetable intakes may consider targeting women who are black or younger or who report high stress, respectively.	5
[41]	After adjustment for confounding variables, participants in the highest quartile of the fruits consumption and vegetables consumption had lower prevalence ratio (PR) for depressive symptoms (PR = 0.76; 95% confidence interval (CI): 0.603–0.974, *p* = 0.042; PR = 0.77; 95% CI: 0.612–0.977, *p* = 0.045) than those in the lowest quartile. Those in the highest quartile of total vegetables and fruits consumption had also a lower PR of depressive symptoms (PR = 0.67; 95% CI: 0.503–0.806, *p* = 0.037) than did those in the lowest quartile.	Higher consumption of vegetables and fruits is significantly associated with a lower risk of depressive symptoms.	5
[42]	Frequent consumption of fruits and vegetables is associated with improved health outcomes, including mental health.	Frequent consumption of fruits and vegetables contributes to slower disablement processes and might be an easily implementable way to improve the overall health of older adults.	6
[43]	Fruit and vegetable consumption predicted an increased cognitive performance in older adults including improved verbal recall, improved delayed verbal recall, improved digit span test performance and improved verbal fluency; the effect of fruit consumption was much stronger than the effect of vegetable consumption. Regarding mental health, fruit consumption was significantly associated with better subjective quality of life and less depressive symptoms; vegetable consumption, however, did not significantly relate to mental health.	Consumption of fruits is associated with both improved cognitive and mental health in older adults from non-Western developing countries, and consumption of vegetables is associated with improved cognitive health only. Increasing fruit and vegetable consumption might be one easy and cost-effective way to improve the overall health and quality of life of older adults in non-Western developing countries.	5
[44]	Consumption of vegetables and fruits in the last 3 days was less likely to be associated with depression (OR 0.11; 95% CI: 0.02–0.45) and subsyndromal depression (OR 0.10; CI 95%: 0.03–0.39).	Findings support the importance for a healthy consumption of vegetables and fruits for the older adult population in Singapore.	5
[45]	Fixed effects regressions show that mental well-being (GHQ-12) responds in a dose-response fashion to increases in both the quantity and the frequency of fruit and vegetables consumed. This relationship is robust to the use of subjective well-being (life satisfaction) instead of mental well-being. Increasing one’s consumption of fruit and vegetables by one portion (on a day where at least one portion is consumed) leads to a 0.133-unit increase in mental well-being (*p* < 0.01).	Persuading people to consume more fruits and vegetables may not only benefit their physical health in the long-run, but also their mental well-being in the short-run.	6
[35]	Results of generalized estimating equations predicting mental-health-related quality of life indicated that more frequent fruit consumption (*p* = 0.485) was not, but more frequent vegetable consumption (*p* = 0.027) was in the fully adjusted model associated with greater mental health-related quality of life. Fruit and vegetable consumption (*p* = 0.033) was associated with greater mental-health-related quality of life only in the unadjusted model. More frequent fruit (*p* = 0.566 and *p* = 0.751, respectively), vegetable (*p* = 0.173 and *p* = 0.399), and fruit and vegetable consumption (*p* = 0.252 and *p* = 0.634, respectively) did not significantly reduce the risk of major depression and generalized anxiety disorder.	The study did not find evidence that more frequent fruit and vegetable consumption was associated with mental-health-related quality of life, depression, and anxiety. However, more frequent vegetable consumption was associated with greater mental-health-related quality of life.	5
[46]	Inverse association was found between mental distress and higher intake of fruits (β = −0.64, 95% CI: −0.89 to −0.39; *p* < 0.001), vegetables (β = − 0.39, 95% CI: −0.65 to −0.13; *p* < 0.003).	Study suggests beneficial association of Mediterranean diet and its elements (including fruit and vegetables intake) and overall mental health, offering important implications for public health provisions.	5
[47]	After adjusting for potential confounders such as age, body-mass-index (BMI), parity, educational status, occupation, marital status, household hunger scale and household asset index, there was an increasing trend across terciles of vegetable intake in the past month for the HR-QoL (*p* = 0.0003), mental health (MH) (*p* = 0.001) domain of the SF-36 and role emotional (*p* < 0.0001) domain of the SF-36. The multivariate model results a significant increasing trend in the adjusted mean scores of the HR-QoL (*p* = 0.04), MH (*p* = 0.001) as well as 4 subscales of the SF-36 [role-physical (*p* = 0.02), role-emotional (*p* = 0.05), emotional well-being (*p* = 0.002) and vitality (*p* < 0.0001)] across terciles of the vegetable variety score.	Results suggest a potential beneficial role of high vegetable intake and consumption of more varied vegetables on HR-QoL.	3
[48]	After adjustment for confounding variables, the participants in the lower quartiles of total fruit and vegetables, total vegetables, total fruits, citrus, other fruits and green leafy vegetables intake were more likely to experience depression compared to those in the higher quartiles (*p* < 0.03).	Lower intake of total fruit and vegetables and some of its specific subgroups might be associated with depression. The findings support encouragement of fruit and vegetables consumption as part of a healthy diet and highlight the importance of fruit and vegetables consumption and a number of their subgroups in mitigating the chance of depression.	7
[49]	Mixed linear models showed that higher baseline levels of psychological well-being were associated with more fruit and vegetable consumption at baseline (β = 0.05, 95% CI: 0.02–0.08) and that fruit and vegetable consumption declined across time (β = 0.01, 95% CI: 0.02–0.004). Psychological well-being interacted significantly with time such that individuals with higher baseline psychological well-being had slower declines in fruit and vegetable consumption (β = 0.01, 95% CI: 0.01–0.02). Among individuals who initially met recommendations to consume 5 or more servings of fruits and vegetables, higher baseline psychological well-being was associated with 11% reduced risk of falling below recommended levels during follow-up (hazard ratio = 0.89; 95% CI: 0.83–0.95).	Psychological well-being may be a precursor to healthy behaviors such as eating a diet rich in fruits and vegetables. Analyses also considered the likelihood of reverse causality.	6
[50]	Controlling for covariates, raw fruit and vegetable intake (FVI) predicted reduced depressive symptoms and higher positive mood, life satisfaction, and flourishing; processed FVI only predicted higher positive mood. The top 10 raw foods related to better mental health were carrots, bananas, apples, dark leafy greens like spinach, grapefruit, lettuce, citrus fruits, fresh berries, cucumber, and kiwifruit.	Raw FVI, but not processed FVI, significantly predicted higher mental health outcomes when controlling for the covariates. Applications include recommending the consumption of raw fruits and vegetables to maximize mental health benefits.	2
[51]	Individuals who were depressed at both times points had the highest proportion who failed to consume any fruit (31%) or vegetables (42%) on the previous day. Fruit and vegetable consumption did not predict of adult depression in fully adjusted models.	Cross sectional associations existed for diet and adolescent depression only. For adult depression association was subsequently attenuated on adjustment for other relevant factors.	5
[52]	Happiness was linearly associated with total fruit and vegetable intakes (*p* = 0.002).	Maintaining good nutrition and increasing fruit and vegetable consumption may be important for psychological health of older people.	4
[53]	Subjects who reported to consume a greater amount of fruit were associated with a lower risk of depression (OR 0.46; 95% CI: 0.26–0.84, *p* = 0.011) after adjustment for many possible confounders. Similar results were obtained for women, while no statistically significant differences emerged for men.	Diet rich in olive oil and fruit, characteristics of Mediterranean diet, may protect against the development of depressive symptoms in older age.	4
[54]	Women in the top quintile of fruit intake, compared with those in the bottom quintile, had 57%, 50%, and 60% lower odds of depression, anxiety, and psychological distress. Consumption of vegetables was significantly associated with lower odds of depression (OR 0.65; 95% CI: 0.46–0.93) in women and lower odds of anxiety (OR 0.43; 95% CI: 0.22–0.87) in men. After adjustment for potential confounders, women in the highest quintile of fruit and vegetables intake, compared with those in the bottom quintile, had significantly lower odds of depression (OR 0.55; 95% CI: 0.37–0.80) and psychological distress (OR 0.60; 95% CI: 0.40–0.90). High intake of total fruit and vegetables was associated with lower odds of psychological distress (OR 0.42; 95% CI: 0.21–0.81) in men.	There was a significant inverse associations between high intake of fruit with depression, anxiety, and psychological distress in Iranian women. High consumption of vegetables was also associated with lower risk of depression and anxiety, in women and men. In addition, high intake of total fruit and vegetable was associated with lower odds of depression and psychological distress in women and men.	6
[55]	Vegetable intake were associated with increased sleep quality, which in turn was associated with increased overall quality of life (*p* < 0.05).	Results suggest possible relationships among the multiple health behaviors and their associations with overall well-being.	3
[56]	Perceived ambiguity and cancer fatalism were negatively associated with fruit and vegetables consumption (*p* < 0.001) whereas health-related self-efficacy was positively associated with fruit and vegetables consumption (b = 0.34, *p* < 0.001).	Individual choice influences fruit and vegetables consumption, but external control beliefs, as measured by perceived ambiguity of cancer prevention recommendations and cancer fatalism, and internal control beliefs, as measured by health-related self-efficacy, may be important inputs to these decisions.	7
[57]	In India, those who consumed less than five servings of vegetables were respectively 41% (AOR = 1.41; 95% CI: 0.60–3.33) and 57% (AOR = 1.57; 95% CI: 0.93–2.64) more likely to report severe-extreme and mild-moderate depression during past 30 days compared to those who consumed five servings a day. Regarding fruit consumption, compared to those who consumed five servings a day, the odds of severe-extreme and mild-moderate self-reported depression were respectively 3.5 times (AOR = 3.48; 95% CI: 1.216–10.01) and 45% (AOR = 1.44; 95% CI: 0.89–2.32) higher in Bangladesh, and 2.9 times (AOR = 2.92; 95% CI: 1.12–7.64) and 42% higher (AOR = 1.41; 95% CI: 0.89–2.24) in Nepal compared to those who consumed less than five servings a day during last 30 days.	Daily intake of less than five servings of fruit and vegetables was associated with higher odds of depression. Nutrition programs aimed at promoting fruit and vegetables consumption might prove beneficial to reduce the prevalence of depression in south Asian population.	5
[58]	Baseline fruit and vegetable consumption considered separately or combined, was associated with a lower prevalence of psychological distress even after adjustment for sociodemographic characteristics and lifestyle risk factors. Baseline fruit and vegetable consumption, measured separately or combined, was associated with a lower incidence of psychological distress in minimally adjusted models. Most of these associations remained significant at medium levels of intake but were no longer significant at the highest intake levels in fully adjusted models.	Increasing fruit and vegetable consumption may help reduce psychological distress in middle-aged and older adults.	8
[59]	Results indicate that the amount of fruit and vegetable consumption was positively associated with happiness and inversely associated with depression. Happiness increased with any increase to fruit and vegetable consumption, the strongest increase in the adjusted analysis was with 6 servings of fruit and vegetables, with a coefficient of 0.41. Depressive symptoms decreased with any increase to fruit and vegetable consumption, the strongest decrease in the adjusted analysis was with 6 servings of fruit and vegetables, with a coefficient of −1.04.	Healthier behavior patterns of fruit and vegetable consumption was associated with higher happiness and lower depression scores among university students across 28 countries.	5
[60]	The intake of green vegetables was associated with lower odds of having clinically-relevant levels of depressive symptoms (OR 0.192, *p* = 0.016).	Green vegetables, total FVI showed protective effects regarding clinically-relevant levels of depressive symptoms.	5
[61]	For depression subtypes, statistically significantly positive associations of vegetable consumption and adherence to the 5-a-day recommendation with current unspecified and current melancholic major depressive disorder were found (OR 2.09; 95% CI: 1.08–4.06 and OR 2.51; 95% CI: 1.21–5.21, respectively; multivariable adjusted for demographic and other dietary factors)	There is no consistent association between adherence to dietary recommendations and major depressive disorder or subtypes of depression.	5
[62]	Mean positive affect increased linearly as a function of number of daily servings of fruits and vegetables; the pattern of this relationship did not differ significantly for males and females. This association remained statistically significant after controlling for demographic variables (age, sex, and parent education levels); other diet variables (consumption of sugar containing beverages, coffee or tea, and fat); and other health behaviors (exercise, sleep quality and smoking). Life satisfaction and negative affect were not significantly related to fruit and vegetable consumption.	There is an association of fruits and vegetable consumption with well-being.	3
[63]	Participants in the lowest tertile of fruits and/or vegetables consumption had greater prevalence of depressive symptoms (PR = 1.88; 95% CI: 1.39–2.55) than those in the highest tertile. This association was stronger with fruits (PR = 1.92; 95% CI: 1.46–2.53) than vegetables (PR = 1.42; 95% CI: 1.05–1.93) alone.	An inverse relationship between consumption of fruits and/or vegetables and depressive symptoms was concluded. There is a need to implement strategies to promote better diet patterns with potential impact on mental health.	6
[64]	High fruit or high vegetable consumption alone (>5 times/week) was not significantly associated with new depressive symptoms. Combining high fruit (OR 0.61; 95% CI: 0.41–0.89), vegetable (OR 0.49; 95% CI: 0.26–0.93) or fruit and vegetable (OR 0.39; 95% CI: 0.20–0.77) consumption with high leisure-time physical activity (LTPA) reduced the likelihood of developing subsequent new depressive symptoms beyond LPTA alone.	The simultaneous presence of several good lifestyle habits (high fruit and vegetable consumption with high leisure-time physical activity) increases the beneficial effect of reducing the risk of developing depressive symptoms in older adults. Thus, older adults are encouraged to have as many good lifestyle habits as possible to reduce the risk of depressive symptoms.	7
[65]	Measure of happiness was positively associated with the amount of fruit and vegetable consumption (*p* = 0.02, and 0.045 respectively). Students who ate breakfast every day, more than 8 servings of fruit and vegetables daily, and had 3 meals in addition to 1–2 snacks per day had the highest happiness score.	Healthier behavior pattern (including fruit and vegetable consumption) was associated with higher happiness scores among medical students.	3
[66]	Increased fruit and vegetable consumption was predictive of increased happiness, life satisfaction, and well-being. They were up to 0.24 life-satisfaction points (for an increase of 8 portions a day), which is equal in size to the psychological gain of moving from unemployment to employment. Improvements occurred within 24 months.	Eating certain foods is a form of investment in future happiness and well-being. The implications of fruit and vegetable consumption are estimated to be substantial and to operate within the space of 2 years.	5
[67]	In males, anxiety was correlated with lower daily intakes of fruits and vegetables (r = −0.216, *p* = 0.013).	A strict plant-based diet does not appear to negatively impact mood.	2
[68]	Fruit and vegetables consumption predicted greater eudaemonic well-being, curiosity, and creativity at the between- and within-person levels. Young adults who ate more fruit and vegetables reported higher average eudaemonic well-being, more intense feelings of curiosity, and greater creativity compared with young adults who ate less fruit and vegetables. On days when young adults ate more fruit and vegetables, they reported greater eudaemonic well-being, curiosity, and creativity compared with days when they ate less fruit and vegetables. Fruit and vegetables consumption also predicted higher positive effects, which mostly did not account for the associations between fruit and vegetables and the other well-being variables. Lagged data analyses showed no carry-over effects of fruit and vegetables consumption onto next-day well-being (or vice versa).	Fruit and vegetables consumption may be related to a broader range of well-being states than signal human flourishing in early adulthood.	4
[69]	Fruit and vegetable consumption at each cycle was inversely associated with next-cycle depression (β = −0.03, 95% CI: −0.05 to −0.01, *p* < 0.01) and psychological distress (β = −0.03, 95% CI: −0.05 to −0.02, *p* < 0.0001). However, once models were adjusted for other health-related factors, these associations were attenuated (β = −0.01, 95% CI: −0.04 to 0.02, *p* = 0.55; β = −0.00, 95% CI: −0.03 to 0.02, *p* = 0.78 for models predicting depression and distress, respectively).	Findings suggest that relations between fruit and vegetable intake, other health-related behaviors and depression are complex.	8
[70]	Significant associations were found between vegetables intake (green salad) and physical health days for Hispanics and fruit and vegetables intake (green salad, fruit, and fruit juice) and self-reported health for Chinese.	There is a need to promote healthy living behaviors among aging NYC racial/ethnic populations	5
[71]	Men who experienced mild to moderate levels of stress were less likely to consume vegetables and fruit (*p* < 0.05) compared with their unstressed counterparts. The trend analysis results indicated significant dose–response patterns in the relationship between stress level and consumption of vegetables and fruit (negative trend) (adjusted OR 0.50; 95% CI: 0.48–0.87; *p* < 0.05). For female students significant dose–response trend was found in the relationship between stress levels and the consumption of vegetables and fruit (both negative trends) (*p* < 0.01).	There is a difference in food selection patterns between stressed male and female students, with stress being a more significant predictor of unhealthy food selection among male students.	4
[72]	Consumers fulfilling the 5-a-day recommendation had lower odds of being highly or moderately distressed than individuals consuming less fruit and vegetables (moderate vs. low distress: OR 0.82; 95% CI: 0.69–0.97; high vs. low distress: OR 0.55; 95% CI: 0.41–0.75).	Daily intake of 5 servings of fruit and vegetable was associated with lower psychological distress.	5
[73]	For females fresh fruits (−0.085; *p* < 0.001), salad/raw vegetables (−0.048; *p* < 0.001), cooked vegetables (−0.061; *p* < 0.001) intake were respectively negatively associated with Perceived Stress Score. For both sexes, consuming fresh fruits (−0.111; *p* < 0.001 for females; −0.074; *p* = 0.047 for males), salads (−0.071; *p* < 0.001 for females; −0.091; *p* = 0.014 for males), cooked vegetables (−0.072; *p* < 0.001 for females; −0.089; *p* = 0.017 for males) was significantly negatively associated with perceived stress and depressive symptoms scores.	The associations between consuming ‘healthy’ foods and lower depressive symptoms and perceived stress among male and female students in three UK countries suggest that interventions to reduce depressive symptoms and stress among students could also result in the consumption of healthier foods and/or vice versa.	5
[74]	Analysis showed reduced odds of depressive symptoms OR 0.86 (95% CI: 0.79–0.95, *p* = 0.001) among women who ate ⩾ 2 of fruit/day and OR 0.79 (95% CI: 0.67–0.93, *p* = 0.007) among women who ate ⩾ 5 vegetables/day, even after adjustment for several factors including smoking, alcohol, body mass index, physical activity, marital status, education, energy, fish intake, and comorbidities.	Increasing fruit consumption may be one important factor for reducing both the prevalence and incidence of depressive symptoms in mid-age women.	6
[75]	Higher Beck Depression Inventory scores correlated with lower fruit and vegetable consumption (r = −0.20, *p* = 0.006). Women reporting a history of treatment for depression showed lower levels of fruit and vegetable consumption (mean daily servings = 2.0 [1.3] vs. 2.5 [1.3], respectively, *p* = 0.03). Participants reporting current antidepressant use (versus non-users) did not differ on dietary habits.	Fruit and vegetable consumption partially mediated associations between depression and time to cardiovascular disease events.	6
[37]	Depressive symptoms were not associated with fruit and vegetables intake (*p* > 0.05).	Future studies should explore the mechanisms linking the identified associations between depressive symptoms and dietary intake, such as the role of emotional eating.	6
[76]	After adjustment for potential confounders, the AHEI score was inversely associated with recurrent depressive symptoms in a dose-response fashion in women (*p* = 0.001; for 1 SD in AHEI score; OR 0.59; 95% CI: 0.47–0.75) but not in men, while among its components vegetable and fruit intake were significant.	Poor diet may be a risk factor for future depression in women.	6
[38]	No significant associations were found between prevalence of distress and vegetable and/or fruit intake (*p* = 0.911 for males; *p* = 0.908 for females).	Psychological distress is not associated with reduced intake fruit and vegetables.	5
[77]	Greater fruit and vegetable intake was significantly associated with lower odds of depression (OR 0.72; 95% CI: 0.71–0.75)—for all 5 waves. Perceived poor mental health status and previous diagnosis of a mood disorder and anxiety disorder also demonstrated statistically significant inverse associations with fruit and vegetable intake (all *p* < 0.05). In the first wave, greater fruit and vegetable intake was significantly associated with lower odds of depression (OR 0.85; 95% CI: 0.78–0.92). A combined estimate of all 5 waves demonstrated similar results (OR 0.72; 95% CI: 0.71–0.75). Relative to those with the lowest fruit and vegetable intake, those with the greatest fruit and vegetable intake also had significantly lower odds of suffering from distress (OR 0.87; 95% CI: 0.78–0.98). These results were consistent across other waves. Perceived poor mental health status and previous diagnosis of a mood disorder and anxiety disorder also demonstrated statistically significant inverse associations with fruit and vegetable intake (all *p* < 0.05).	Findings suggest a potentially important role of a healthy diet in the prevention of depression and anxiety.	5
[78]	There was no significant difference (*p* > 0.05) of intake of vegetable products and dishes in women with depression compared with women without depression. The regression model indicated among others that increased intakes per kilojoule of vegetables (*p* = 0.015) are associated with lower odds of having depression.	The results confirm a collective effect of diet on mood.	6
[79]	After adjustments for potentially confounding factors, the odds ratios of having mild and severe depressive symptoms by increasing levels of tomatoes/tomato products were 1.00, 0.54, and 0.48 (*p* <0.01). No relationship was observed between intake of other kinds of vegetables and depressive symptoms.	Tomato-rich diet is independently related to lower prevalence of depressive symptoms and may have a beneficial effect on the prevention of depressive symptoms.	6
[80]	Dietary intake of inter alia fruits and vegetables was significantly higher in the low-stress group than in high-stress group. There was an inverse association between stress level and intake of fruits and vegetables (OR 0.83; 95% CI: 0.76–0.90).	The results showed a significant positive association between dietary intake and stress. There must be a special attention to dietary intake in stress management program of high-stress individuals, and in dietary recommendations, psychologic aspects should be considered.	5
[81]	Analyses of same-day within-person associations revealed that on days when young adults experienced greater positive affect, they reported eating more servings of fruit (*p* = 0.002) and vegetables (*p* < 0.001). Results of lagged analysis showed that fruits and vegetables predicted improvements in positive affect the next day, suggesting that healthy foods were driving affective experiences and not vice versa. Meaningful changes in positive affect were observed with the daily consumption of approximately 7–8 servings of fruit or vegetables.	Eating fruit and vegetables may promote emotional well-being among healthy young adults.	3
[82]	In cross-sectional data, happiness and mental health rise in an approximately dose–response way with the number of daily portions of fruit and vegetables. Well-being peaks at approximately 7 portions per day. It was documented for seven measures of well-being (life satisfaction, WEMWBS mental well-being, GHQ mental disorders, self-reported health, happiness, nervousness, and feeling low). The pattern is robust to adjustment for a large number of other demographic, social and economic variables.	There is a positive association between eating fruit and vegetables and having high mental well-being.	4
[83]	Compared to the regional nutrition survey data, a greater proportion of study participants (the members of Mood Disorders Association of British Columbia) consumed fewer of the recommended servings of vegetables and fruits (*p* < 0.05).	The adults with mood disorders could benefit from nutritional interventions to improve diet quality.	4
[84]	Fruit and vegetable consumption was lower in depressed individuals, than in comparison individuals, that remained significant in multivariable models.	These results may indicate the importance of components of fruits and vegetables, including antioxidants, rather than dietary supplements.	6
[85]	In a regression model that controlled for demographic, socio-economic, lifestyle and disease/health-related variables but not cognitive status, both fruits (OR 0.66; 95% CI: 0.45–0.98, *p* = 0.038) and vegetables (OR 0.38; 95% CI: 0.17–0.86, *p* = 0.021) were protective against depressive symptoms 4 years later. When the same regression model was also adjusted for cognitive status, only vegetables (OR 0.40, 95% CI: 0.17–0.95, *p* = 0.039) were protective against depressive symptoms.	More frequent consumption of vegetables seems to be protective against depressive symptoms in the elderly.	7
[36]	Patients who ate fruit more frequently tended to have a better quality of life.	There is an association between food pattern and quality of life in this population.	2
[39]	Neither mental nor physical health was associated with fruit and vegetable intake (*p* < 0.05).	Fruit and vegetable consumption may be too specific to represent an individual’s overall diet.	4
[86]	Depressive symptoms were related to a lower consumption of vegetables/fruit.	Depressive symptoms may affect unhealthy food choices. The relations between negative emotions such as depressive symptoms and food consumption are most likely bidirectional: food consumed affects mood and mood affects food choices.	6
[87]	The consumption of vegetables is more prevalent among people with low or moderate depression than those with severe depression (*p* < 0.05).	Unhealthy dietary choices seems to promote depression. Efforts to lower the prevalence of depression in the elderly should target on the factors such as dietary habits.	6
[88]	In men, attempters (n = 92) had a high odds of low consumption of vegetables (OR 2.47, 95% CI: 1.19–5.15). In women, attempters (n = 275) had a high odds of insufficient fruit consumption (OR 2.36, 95% CI: 1.15–4.85).	The data suggest that fruits and vegetables were significantly under-consumed in adults who had ever attempted suicide.	6
[89]	For male students, none of the food consumption groups were associated with perceived stress or depressive symptoms. In females, perceived stress and depressive symptoms were associated with less frequent consumption of fruits/vegetables.	Consistent associations were observed between unhealthy food consumption and depressive symptoms and perceived stress among female students from three European countries, but not among male students. Efforts to reduce depressive symptoms and stress among female students may also lead to the consumption of healthier foods and/or vice-versa.	4
[90]	Single mothers had lower intake of fruits and vegetables and lower self-worth compared to the married and cohabiting mothers, controlling for age, education and BMI.	A lower sense of self-worth and lower intake of fruit and vegetables in single mothers could be seen in the context of the social disadvantages and less social support.	6
[91]	A high level of dispositional optimism was associated with higher intakes of fruit (*p* = 0.01), and vegetables (*p* = 0.01), independently from age, education, living arrangement, self-rated health, cardiovascular disease, diabetes mellitus, cancer, and body mass index, as well as total energy intake.	Dispositional optimism in elderly men is associated with healthy lifestyle and dietary habits.	7
[92]	Stepwise logistic regression models found that frequency of consumption of fresh fruit had apparently independent effects on perceived stress, whereas the intake level of fresh fruit was significantly associated with depression.	The link between food consumption frequency, perceived stress and depression suggests that diet intervention may be considered a mediate strategy integrated in psychology prevention program among normal population of the college.	5
[93]	Both women and men above the upper quartile for optimism more often ate fresh vegetables and salads (women 6%/men 57%), berries (23%/9%), fruit (67%/42%), than those below the lower quartile (56%/31%, 14%/5%, and 52%/26%, respectively) with women in higher proportion than in men in each case.	Lack of optimism is associated with a cluster of unhealthy dietary and other habits.	7
[94]	Women having poor mental health were less likely than their healthier counterparts to report consuming fresh vegetables, fresh fruits on a daily basis. Men having poor mental health reported consuming less frequently fresh fruits. These results remained statistically significant in the fully adjusted model.	The results suggest that poor mental health is associated with unhealthy food habits.	5
[95]	The mood of females was significantly better when more fruit/vegetables were consumed (F(2,157) = 12.39, *p* < 0.001). There was no association between the amount consumed by males and scores on the GHQ [F(2,163) = 1.09, NS]. In females a lower intake of fruit/vegetables was associated with higher levels of anxiety (F(2,157) = 8.78, *p* < 0.002), a relationship not found in males (F(2,163) = 0.29, NS). Females who consumed greater amounts of fruit/vegetables were less depressed [F(2,157) = 12.77, *p* < 0.0001], although there was no such relationship in males (F(2,163) = 1.23, NS).	Eating large amounts of fruit and vegetables were less likely to be anxious or depressed; the relationship existed irrespective of age and social background.	4

* total score for the Newcastle–Ottawa Scale (NOS) is attributed to a following categories: very high risk of bias (0–3 NOS points), high risk of bias (4–6 NOS points), and low risk of bias (7–9 NOS points) [23].

## Supplementary Materials

The following are available online at https://www.mdpi.com/2072-6643/12/1/115/s1, Table S1: Full electronic search strategy applied for PubMed and Web of Science databases, Table S2: List of studies included to the systematic review (in alphabetic order), Table S3: Characteristics of the study participants for the studies included to the systematic review, Table S4: Detailed results of the quality assessment based on the total score for the Newcastle–Ottawa Scale for categories of selection, comparability and exposure/outcome.
