# Supplementary material for: Fruit and Vegetable Intake and Mental Health in Adults: A Systematic Review
Source: Nutrients. 2020 Jan 1;12(1):115. doi: 10.3390/nu12010115 (PMC7019743; doi:10.3390/nu12010115)
Supplement: Supplementary file 1 [file nutrients-12-00115-s001.pdf]

Review

Fruit and vegetable intake and mental health in adults: a systematic review

Dominika Głąbska, Dominika Guzek, Barbara Groele, Krystyna Gutkowska

Supplementary Table 1. Full electronic search strategy applied for PubMed and Web of Science databases.

Database	The applied full electronic search strategy
PubMed	(((fruit[Title/Abstract] OR fruits[Title/Abstract] OR vegetable[Title/Abstract] OR vegetables[Title/Abstract] OR juice[Title/Abstract] OR juices[Title/Abstract])) AND (mental health[Title/Abstract] OR mental disorder[Title/Abstract] OR mental disorders[Title/Abstract] OR psychological distress[Title/Abstract] OR mood disorders[Title/Abstract] OR depression[Title/Abstract] OR anxiety[Title/Abstract] OR suicide[Title/Abstract] OR suicidal[Title/Abstract] OR well-being[Title/Abstract] OR wellbeing[Title/Abstract] OR quality of life[Title/Abstract] OR self-esteem[Title/Abstract] OR self esteem[Title/Abstract] OR self-efficacy[Title/Abstract] OR self efficacy[Title/Abstract] OR resilience[Title/Abstract] OR empowerment[Title/Abstract] OR life skills[Title/Abstract] OR social participation[Title/Abstract] OR mental capital[Title/Abstract] OR emotional[Title/Abstract] OR psychosocial[Title/Abstract] OR psychology[Title/Abstract] OR psychiatry[Title/Abstract])) NOT (animal NOT (animal AND human)[MeSH Terms])
Web of Science	(TS=(fruit OR fruits OR vegetable OR vegetables OR juice OR juices) AND TS=("mental health" OR "mental disorder" OR "mental disorders" OR "psychological distress" OR "mood disorder" OR depression OR anxiety OR suicide OR suicidal OR well-being OR wellbeing OR "quality of life" OR self-esteem OR "self esteem" OR self-efficacy OR "self efficacy" OR resilience OR empowerment OR "life skills" OR "social participation" OR "mental capital" OR emotional OR psychosocial OR psychology OR psychiatry) NOT TS=(animal NOT (animal AND human)))

Supplementary Table 2. List of studies included to the systematic review (in alphabetic order).

**Studies included**	**Ref.**
Akbaraly, T.N.; Sabia, S.; Shipley, M.J.; Batty, G.D.; Kivimaki, M. Adherence to healthy dietary guidelines and future depressive symptoms: evidence for sex differentials in the Whitehall II study. *Am. J. Clin. Nutr.* **2013**, *97(2)*, 419-427. doi: 10.3945/ajcn.112.041582.	[74]
Azupogo, F.; Seidu, J.A.; Issaka, Y.B. Higher vegetable intake and vegetable variety is associated with a better self-reported health-related quality of life (HR-QoL) in a cross-sectional survey of rural northern Ghanaian women in fertile age. *BMC Public Health.* **2018**, *27;18(1)*, 920. doi: 10.1186/s12889-018-5845-3.	[45]
Baharzadeh, E.; Siassi, F.; Qorbani, M.; Koohdani, F.; Pak, N.; Sotoudeh, G. Fruits and vegetables intake and its subgroups are related to depression: a cross-sectional study from a developing country. *Ann. Gen. Psychiatry* **2018**, *1;17*, 46. doi: 10.1186/s12991-018-0216-0.	[46]
Beezhold, B.; Radnitz, C.; Rinne, A.; DiMatteo, J. Vegans report less stress and anxiety than omnivores. *Nutr. Neurosci.* **2015**, *18(7)*, 289-296. doi: 10.1179/1476830514Y.0000000164.	[65]
Bhattacharyya, M.; Marston, L.; Walters, K.; D'Costa, G.; King, M.; Nazareth, I. Psychological distress, gender and dietary factors in South Asians: a cross-sectional survey. *Public Health Nutr.* **2014**, *17(7)*, 1538-1546. doi: 10.1017/S136898001300147X.	[75]
Bishwajit, G.; O'Leary, D.P.; Ghosh, S.; Sanni, Y.; Shangfeng, T.; Zhanchun, F. Association between depression and fruit and vegetable consumption among adults in South Asia. *BMC Psychiatry.* **2017**, *14;17(1)*, 15. doi: 10.1186/s12888-017-1198-1.	[55]
Blanchflower, D.G.; Oswald, A.J.; Stewart-Brown, S. Is psychological well-being linked to the consumption of fruit and vegetables? *Soc. Indic. Res.* **2013**, *114(3)*, 785-801	[81]
Boehm, J.K.; Soo, J.; Zevon, E.S.; Chen, Y.; Kim, E.S.; Kubzansky, L.D. Longitudinal associations between psychological well-being and the consumption of fruits and vegetables. *Health Psychol.* **2018**, *37(10)*, 959-967. doi: 10.1037/hea0000643.	[47]
Brookie, K.L.; Best, G.I.; Conner, T.S. Intake of Raw Fruits and Vegetables Is Associated With Better Mental Health Than Intake of Processed Fruits and Vegetables. *Front. Psychol.* **2018**, *10;9*, 487. doi: 10.3389/fpsyg.2018.00487.	[48]
Chai, W.; Nigg, C.R.; Pagano, I.S.; Motl, R.W.; Horwath, C.; Dishman, R.K. Associations of quality of life with physical activity, fruit and vegetable consumption, and physical inactivity in a free living, multiethnic population in Hawaii: a longitudinal study. *Int. J. Behav. Nutr. Phys. Act.* **2010**, *22;7*, 83. doi: 10.1186/1479-5868-7-83.	[85]
Chang, M.W.; Tan, A.; Schaffir, J. Relationships between stress, demographics and dietary intake behaviours among low-income pregnant women with overweight or obesity. Public Health Nutr. 2019, 22(6), 1066-1074. doi: 10.1017/S1368980018003385	[38]
Cheng, H.Y.; Shi, Y.X.; Yu, F.N.; Zhao, H.Z.; Zhang, J.H.; Song, M. Association between vegetables and fruits consumption and depressive symptoms in a middle-aged Chinese population: An observational study. *Medicine (Baltimore).* **2019**, *98(18)*, 15374. doi: 10.1097/MD.0000000000015374.	[39]
Chi, S.H.; Wang, J.Y.; Tsai, A.C. Combined association of leisure-time physical activity and fruit and vegetable consumption with depressive symptoms in older Taiwanese: Results of a national cohort study. *Geriatr. Gerontol. Int.* **20 16**, *16(2)*, 244-251. doi: 10.1111/ggi.12459.	[62]
Conner, T.S.; Brookie, K.L.; Richardson, A.C.; Polak, M.A. On carrots and curiosity: eating fruit and vegetables is associated with greater flourishing in daily life. *Br. J. Health Psychol.* **2015**, *20(2)*, 413-427. doi: 10.1111/bjhp.12113.	[66]
Cook, R.; Benton, D. The relationship between diet and mental-health. *Person. Individ. Diff.* **1993**, *14(3)*, 397-403.	[95]
Davison, K.M.; Kaplan, B.J. Food intake and blood cholesterol levels of community-based adults with mood disorders. *BMC Psychiatry* **2012**, *14;12*, 10. doi: 10.1186/1471-244X-12-10.	[82]
El Ansari, W.; Adetunji, H.; Oskrochi, R. Food and mental health: relationship between food and perceived stress and depressive symptoms among university students in the United Kingdom. *Cent. Eur. J. Public Health.* **2014**, *22(2)*, 90-97.	[71]
Elfhag, K.; Rasmussen, F. Food consumption, eating behaviour and self-esteem among single v. married and cohabiting mothers and their 12-year-old children. *Public Health Nutr.* **2008**, *11(9)*,	[90]

**Studies included**	**Ref.**
934-939. doi: 10.1017/S1368980008002449.	
Gehlich, K.H.; Beller, J.; Lange-Asschenfeldt, B.; Köcher, W.; Meinke, M.C.; Lademann, J. Consumption of fruits and vegetables: improved physical health, mental health, physical functioning and cognitive health in older adults from 11 European countries. *Aging. Ment. Health.* **2019**, *7*, 1-8. doi: 10.1080/13607863.2019.1571011.	[40]
Gehlich, K.H.; Beller, J.; Lange-Asschenfeldt, B.; Köcher, W.; Meinke, M.C.; Lademann, J. Fruit and vegetable consumption is associated with improved mental and cognitive health in older adults from non-Western developing countries. *Public Health Nutr.* **2019**, *22(4),* 689-696. doi: 10.1017/S1368980018002525.	[41]
Giltay, E.J.; Geleijnse, J.M.; Zitman, F.G.; Buijsse, B.; Kromhout, D. Lifestyle and dietary correlates of dispositional optimism in men: The Zutphen Elderly Study. *J. Psychosom. Res.* **2007**, *63(5),* 483-490.	[91]
Goh, C.M.J.; Abdin, E.; Jeyagurunathan, A.; Shafie, S.; Sambasivam, R.; Zhang, Y.J.; Vaingankar, J.A.; Chong, S.A.; Subramaniam, M. Exploring Singapore's consumption of local fish, vegetables and fruits, meat and problematic alcohol use as risk factors of depression and subsyndromal depression in older adults. *BMC Geriatr.* **2019**, *10;19(1),* 161. doi: 10.1186/s12877-019-1178-z.	[42]
Hoare, E.; Hockey, M.; Ruusunen, A.; Jacka, F.N. Does Fruit and Vegetable Consumption During Adolescence Predict Adult Depression? A Longitudinal Study of US Adolescents. Front. *Psychiatry* **2018**, *13;9,* 581. doi: 10.3389/fpsyt.2018.00581.	[49]
Jyväkorpi, S.K.; Urtamo, A.; Pitkala, K.H.; Strandberg, T. E. Happiness of the oldest-old men is associated with fruit and vegetable intakes. *Eur. Geriatr. Med.* **2018**, *9(5),* 687-690.	[50]
Kelloniemi, H.; Ek, E.; Laitinen, J. Optimism, dietary habits, body mass index and smoking among young Finnish adults. *Appetite* **2005**, *45(2),* 169-176.	[93]
Kingsbury, M.; Dupuis, G.; Jacka, F.; Roy-Gagnon, M.H.; McMartin, S.E.; Colman, I. Associations between fruit and vegetable consumption and depressive symptoms: evidence from a national Canadian longitudinal survey. *J. Epidemiol. Community Health.* **2016**, *70(2),* 155-161. doi: 10.1136/jech-2015-205858.	[67]
Konttinen, H.; Männistö, S.; Sarlio-Lähteenkorva, S.; Silventoinen, K.; Haukkala, A. Emotional eating, depressive symptoms and self-reported food consumption. A population-based study. *Appetite* **2010**, *54(3),* 473-479. doi: 10.1016/j.appet.2010.01.014.	[86]
Kwon, S.C.; Wyatt, L.C.; Kranick, J.A.; Islam, N.S.; Devia, C.; Horowitz, C.; Trinh-Shevrin, C. Physical activity, fruit and vegetable intake, and health-related quality of life among older Chinese, Hispanics, and Blacks in New York City. *Am. J. Public Health.* **2015**, *105,* 544-552. doi: 10.2105/AJPH.2015.302653.	[68]
Lesani, A.; Mohammadpoorasl, A.; Javadi, M.; Esfeh, J.M.; Fakhari, A. Eating breakfast, fruit and vegetable intake and their relation with happiness in college students. *Eat Weight Disord.* **2016**, *21(4),* 645-651.	[63]
Li, Y.; Zhang, J.; McKeown, R.E. Cross-sectional assessment of diet quality in individuals with a lifetime history of attempted suicide. *Psychiatry Res.* **2009**, *30;165(1-2),* 111-119. doi:10.1016/j.psychres.2007.09.004.	[88]
Liu, C.; Xie, B.; Chou, C.P.; Koprowski, C.; Zhou, D.; Palmer, P.; Sun, P.; Guo, Q.; Duan, L.; Sun, X.; Anderson-Johnson, C. Perceived stress, depression and food consumption frequency in the college students of China Seven Cities. *Physiol. Behav.* **2007**, *23;92(4),* 748-754.	[92]
Mamplekou, E.; Bountziouka, V.; Psaltopoulou, T.; Zeimbekis, A.; Tsakoundakis, N.; Papaerakleous, N.; Gotsis, E.; Metallinos, G.; Pounis, G.; Polychronopoulos, E.; Lionis, C.; Panagiotakos, D. Urban environment, physical inactivity and unhealthy dietary habits correlate to depression among elderly living in eastern Mediterranean islands: the MEDIS (MEDiterranean ISlands Elderly) study. *J. Nutr. Health Aging* **2010**, *14(6),* 449-455.	[87]
McMartin, S.E.; Jacka, F.N.; Colman, I. The association between fruit and vegetable consumption and mental health disorders: evidence from five waves of a national survey of Canadians. *Prev. Med.* **2013**, *56(3-4),* 225-230. doi: 10.1016/j.ypmed.2012.12.016.	[76]
Meyer, B.J.; Kolanu, N.; Griffiths, D.A.; Grounds, B.; Howe, P.R.; Kreis, I.A. Food groups and	[77]

**Studies included**	**Ref.**
fatty acids associated with self-reported depression: an analysis from the Australian National Nutrition and Health Surveys. *Nutrition* **2013**, *29(7-8)*, 1042-1047. doi: 10.1016/j.nut.2013.02.006.	
Mihrshahi, S.; Dobson, A.J.; Mishra, G.D. Fruit and vegetable consumption and prevalence and incidence of depressive symptoms in mid-age women: results from the Australian longitudinal study on women's health. *Eur. J. Clin. Nutr.* **2015**, *69(5)*, 585-591. doi: 10.1038/ejcn.2014.222.	[72]
Mikolajczyk, R.T.; El Ansari, W.; Maxwell, A.E. Food consumption frequency and perceived stress and depressive symptoms among students in three European countries. *Nutr. J.* **2009**, *15;8*, 31. doi: 10.1186/1475-2891-8-31.	[89]
Mujcic, R.; Oswald, J.A. Evolution of Well-Being and Happiness After Increases in Consumption of Fruit and Vegetables. *Am. J. Public Health.* **2016**, *106(8)*, 1504-1510. doi: 10.2105/AJPH.2016.303260.	[64]
Nguyen, B.; Ding, D.; Mihrshahi, S. Fruit and vegetable consumption and psychological distress: cross-sectional and longitudinal analyses based on a large Australian sample. *BMJ Open.* **2017**, *15;7(3)*, 014201. doi: 10.1136/bmjopen-2016-014201.	[56]
Niu, K.; Guo, H.; Kakizaki, M.; Cui, Y.; Ohmori-Matsuda, K.; Guan, L.; Hozawa, A.; Kuriyama, S.; Tsuboya, T.; Ohrui, T.; Furukawa, K.; Arai, H.; Tsuji, I.; Nagatomi, R. A tomato-rich diet is related to depressive symptoms among an elderly population aged 70 years and over: a population-based, cross-sectional analysis. *J. Affect. Disord.* **2013**, *10;144(1-2)*, 165-170. doi: 10.1016/j.jad.2012.04.040.	[78]
Ocean, N.; Howley, P.; Ensor, J. Lettuce be happy: A longitudinal UK study on the relationship between fruit and vegetable consumption and well-being. *Soc. Sci. Med.* **2019**, *222*, 335-345. doi: 10.1016/j.socscimed.2018.12.017.	[43]
Pagliai, G.; Sofi, F.; Vannetti, F.; Caiani, S.; Pasquini, G.; Lova, R. Molino; Cecchi, F.; Sorbi, S.; Macchi, C. 2018. Mediterranean diet, food consumption and risk of late-life depression: The Mugello Study. *J. Nutr. Health Aging* **2018**, *2(5)*, 569-574.	[51]
Papier, K.; Ahmed, F.; Lee, P.; Wiseman, J. Stress and dietary behaviour among first-year university students in Australia: sex differences. *Nutrition* **2015**, *31(2)*, 324-330. doi: 10.1016/j.nut.2014.08.004.	[69]
Payne, M.E.; Steck, S.E.; George, R.R.; Steffens, D.C. Fruit, vegetable, and antioxidant intakes are lower in older adults with depression. *J. Acad. Nutr. Diet.* **2012**, *112(12)*, 2022-2027. doi: 10.1016/j.jand.2012.08.026.	[83]
Peltzer, K.; Pengpid, S. Dietary consumption and happiness and depression among university students: A cross-national survey. *J. Psychol. Afr.* **2017**, *27(4)*, 372-377.	[57]
Pengpid, S.; Peltzer, K. Association between fruit/vegetable consumption and mental-health-related quality of life, major depression, and generalized anxiety disorder: A longitudinal study in Thailand. *Iran. J. Psychiatry Behav. Sci.* **2019**, *13(2)*, 88246	[35]
Ribeiro, S.M.L.; Malmstrom, T.K.; Morley, J.E.; Miller, D.K. Fruit and vegetable intake, physical activity, and depressive symptoms in the African American Health (AAH) study. ***J. Affect. Disord.*** **2017**, *1;220*, 31-37. doi: 10.1016/j.jad.2017.05.038.	[58]
Richard, A.; Rohrmann, S.; Vandeleur, C.L.; Lasserre, A.M.; Strippoli, M.F.; Eichholzer, M.; Glaus, J.; Marques-Vidal, P.; Vollenweider, P.; Preisig, M. Adherence to dietary recommendations is not associated with depression in two Swiss population-based samples. *Psychiatry Res.* **2017**, *252*, 310-318. doi: 10.1016/j.psychres.2017.03.017.	[59]
Richard, A.; Rohrmann, S.; Vandeleur, C.L.; Mohler-Kuo, M.; Eichholzer, M. Associations between fruit and vegetable consumption and psychological distress: results from a population-based study. *BMC Psychiatry* **2015**, *1;15*, 213. doi: 10.1186/s12888-015-0597-4.	[70]
Roohafza, H.; Sarrafzadegan, N.; Sadeghi, M.; Rafieian-Kopaei, M.; Sajjadi, F.; Khosravi-Boroujeni, H. The association between stress levels and food consumption among Iranian population. *Arch. Iran Med.* **2013**, *16(3)*, 145-148. doi: 013163/AIM.005.	[79]
Rutledge, T.; Kenkre, T.S.; Thompson, D.V.; Bittner, V.A.; Whittaker, K.; Eastwood, J.A.; Eteiba, W.; Cornell, C.E.; Krantz, D.S.; Pepine, C.J.; Johnson, B.D.; Handberg, E.M.; Bairey Merz, C.N. Depression, dietary habits, and cardiovascular events among women with suspected myocardial ischemia. *Am. J. Med.* **2014**, *127(9)*, 840-807. doi: 10.1016/j.amjmed.2014.04.011.	[73]

**Studies included**	**Ref.**
Saghafian, F.; Malmir, H.; Saneei, P.; Keshteli, A.H.; Hosseinzadeh-Attar, M.J.; Afshar, H.; Siassi, F.; Esmaillzadeh, A.; Adibi, P. Consumption of fruit and vegetables in relation with psychological disorders in Iranian adults. *Eur. J. Nutr.* **2018**, *57(6),* 2295-2306. doi: 10.1007/s00394-018-1652-y.	[52]
Salvatore, F.P.; Relja, A.; Simunovic Filipcic, I.; Polasek, O.; Kolcic, I. Mediterranean diet and mental distress: 10,001 Dalmatians study. Mediterranean diet and mental distress: 10,001 Dalmatians study. *Br. Food J.* **2019,** *121(6),* 1314-1326.	[44]
Sarlio-Lähteenkorva, S.; Lahelma, E.; Roos, E. Mental health and food habits among employed women and men. *Appetite* **2004**, *42(2),* 151-156.	[94]
Tan, S.L.; Storm, V.; Reinwand, D.A.; Wienert, J.; de Vries, H.; Lippke, S. Understanding the Positive Associations of Sleep, Physical Activity, Fruit and Vegetable Intake as Predictors of Quality of Life and Subjective Health Across Age Groups: A Theory Based, Cross-Sectional Web-Based Study. *Front. Psychol.* **2018**, *18;9*, 977. doi: 10.3389/fpsyg.2018.00977.	[53]
Tsai, A.C.; Chang, T.L.; Chi, S.H. Frequent consumption of vegetables predicts lower risk of depression in older Taiwanese - results of a prospective population-based study. *Public Health Nutr.* **2012**, *15(6),* 1087-1092. doi: 10.1017/S1368980011002977.	[84]
Tung, H.H.; Tseng, L.H.; Wei, J.; Lin, C.H.; Wang, T.J.; Liang, S.Y. Food pattern and quality of life in metabolic syndrome patients who underwent coronary artery bypass grafting in Taiwan. *Eur. J. Cardiovasc Nurs.* **2011**, *10(4),* 205-212. doi: 10.1016/j.ejcnurse.2010.05.004.	[36]
Warner, R.M.; Frye, K; Morrell, J.S; Carey, G. Fruit and Vegetable Intake Predicts Positive Affect. *J. Happiness Stud.* **2017**, *18(3),* 809-826	[60]
Welch, J.D.; Ellis, E.M. Sex Differences in the Association of Perceived Ambiguity, Cancer Fatalism, and Health-Related Self-Efficacy with Fruit and Vegetable Consumption. *J. Health Commun.* **2018**, *23(12),* 984-992. doi: 10.1080/10810730.2018.1534905.	[54]
Whitaker, K.M.; Sharpe, P.A.; Wilcox, S.; Hutto, B.E. Depressive symptoms are associated with dietary intake but not physical activity among overweight and obese women from disadvantaged neighborhoods. *Nutr. Res.* **2014**, *34(4),* 294-301. doi: 10.1016/j.nutres.2014.01.007.	[37]
White, B.A.; Horwath, C.C.; Conner, T.S. Many apples a day keep the blues away--daily experiences of negative and positive affect and food consumption in young adults. *Br. J. Health Psychol.* **2013**, *18(4),* 782-798. doi: 10.1111/bjhp.12021.	[80]
Wolniczak, I.; Cáceres-DelAguila, J.A.; Maguiña, J.L.; Bernabe-Ortiz, A. Fruits and vegetables consumption and depressive symptoms: A population-based study in Peru. *PLoS One.* **2017**, *12;12(10),* 0186379. doi: 10.1371/journal.pone.0186379.	[61]

Supplementary Table 3. Characteristics of the study participants for the studies included to the systematic review.

Ref.	Number of participants (females)	Age (mean with SD/ range)	Inclusion criteria/ Exclusion criteria
[38]	353 (353)	25.7 ± 5.5	Inclusion criteria: Low-income, pregnant, pre-pregnancy BMI ≥25.0, ≥ 18 years, English speaking<br>Exclusion criteria: Hispanic ethnicity
[39]	1,676 (890)	45–59 old years	Inclusion: 45-59 years, attending their health examinations at the Medical Center for Physical Examination, Linyi People's Hospital<br>Exclusion: Missing data, participants with a total energy intake of > 3000 kcal/d
[40]	22,635 (not specified)	64.4 ± 9.3	Inclusion: > 50 years<br>Exclusion: Participants who only participated in one wave of study, missing data
[41]	28,078 (not specified)	63.3 ± 9.6	Inclusion: ≥ 50 years<br>Exclusion: Missing data
[42]	2,565 (1,448)	72.7 ± 9.5	Inclusion criteria: ≥60 years, Singapore citizens and permanent residents at the time of the survey<br>Exclusion criteria: Uncontactable at the time of the survey
[43]	52,194/58,156 (28,864/31,861) [1]	47.1 (15–104)	Inclusion: -<br>Exclusion: < 15 years old
[35]	442 (328) at baseline	51.1 ± 7.4	Inclusion criteria: 35–65 years, temple member, prehypertension and/or prediabetes<br>Exclusion criteria: cardiovascular disease, type 2 diabetes, hypertension, substance abuse, and taking psychotropic medication
[44]	3,392 (2,110)	Not specified for all participants combined	Inclusion criteria: Inhabitants of Island of Korčula and from the City of Split<br>Exclusion criteria: < 18 years
[45]	187 (187)	38.1 ± 11.2	Inclusion: 15–49 years, apparently healthy<br>Exclusion: Lactating, pregnant
[46]	400 (400)	33.9 ± 7.3 [1]	Inclusion: 20–49 years, at least fifth grade elementary education, BMI of 18.5–34.9 kg/m^2<br>Exclusion: Pregnancy and lactation, diagnosis of depression by a psychiatrist within a year prior to the start of the study, taking an antidepressant medication in the past year, use of tobacco or alcohol at least once a week, diabetes, cardiovascular diseases, cancer, hypertension, kidney and liver disease, hyperthyroidism, epilepsy and multiple sclerosis, regular use of any medication or following any special diet
[47]	6,565 (3,639)	Baseline: 65.0 ± 9.8	Inclusion: ≥ 50 years<br>Exclusion: Missing data
[48]	422 (279)	21.6 ± 2.1	Inclusion: New Zealand university students or USA sample gathered using Amazon's Mechanical Turk<br>Exclusion: Missing data, suspected errors in responding
[49]	3,696 (not specified)	Baseline: 15.9 ± 1.7	Inclusion: students 12–18 years<br>Exclusion: Missing data, not completed follow-up data collection
[50]	338 (NA)	88 (82–97)	Inclusion criteria: Oldest-old, home-dwelling men<br>Exclusion criteria: -
[51]	388 (271)	92.7 ± 3.1	Inclusion criteria: 90–99 years old, living in the Mugello area<br>Exclusion criteria: -
[52]	3,362 (1,959) [1]	36.3 ± 7.8 [1]	Inclusion: Working in health centers affiliated with the Isfahan University of Medical Sciences (IUMS)<br>Exclusion: Missing data, under- and over-reporters (energy intake out of the range of 800–4200 kcal/day)

Ref.	Number of participants (females)	Age (mean with SD/ range)	Inclusion criteria/ Exclusion criteria
[53]	790 (497)	50.9	Inclusion: ≥ 20 years, German or Dutch language proficiency, having an interest to reduce cardiac risk behavior in terms of being able to be physically active at least 150 min per week and being able to eat at least five portions of fruit and vegetables a day, no complications and restrictions for physical activity and fruit and vegetable intake, Internet access<br>Exclusion: Missing data
[54]	16,965 (8,737)	45.2 ± 19.7	Inclusion: -<br>Exclusion: -
[55]	14,133 (7,898)	Bangladesh 39.7 ± 15.3,<br>India 39.1 ± 15.3, Nepal 42.6 ± 16.6	Inclusion: ≥ 18 years, residing in non-institutional settings<br>Exclusion: Military reservations, or other non-household living arrangements
[56]	60,404 (32,347)	62.2 ± 10.6	Inclusion: ≥ 45 years<br>Exclusion: At baseline treated for depression/ anxiety in the previous month, and/or taking antidepressant medication for most of the past 4 weeks, and/ or with high/ very high levels of psychological distress [Kessler Psychological Distress Scale (K10) score ≥22]
[57]	18,522 (10,708)	20.9 ± 2.4	Inclusion criteria: 18–30 years, undergraduate students, speaking Arabic, Bahasa, Chinese, French, Lao, Russian, Spanish, Thai, Turkish, or Vietnamese<br>Exclusion criteria: -
[58]	680 (444) in 2007	64.1 ± 4.4 at baseline	Inclusion: Born in 1936–1950, African American, lived Saint Louis, Missouri in 2000–2001<br>Exclusion: Institutionalized, MMSE (Mini-Mental State Examination) < 16
[59]	14,652 (7,130)	≥ 40 years	Inclusion: ≥ 40 years<br>Exclusion: Missing data, cofounders
[60]	1,270 (879)	19.1 ± 1.2	Inclusion criteria: 18–25 years, students of Nutrition or Psychology courses<br>Exclusion criteria: -
[61]	25,848 (13,944)	44.2 ± 17.7	Inclusion: ≥ 18 years, habitual residents of the study setting<br>Exclusion: Missing data [1]
[62]	2,630 (1,168)	≥ 53 years	Inclusion: ≥ 53 year<br>Exclusion: Proxy respondents, depressive symptoms at baseline, missing data, died or lost to follow up
[63]	541 (403)	24.1 ± 4.9	Inclusion: Students of Qazvin University of Medical Sciences in Iran<br>Exclusion: -
[64]	12,385 (not specified)	15–93 years	Inclusion: ≥ 15 years<br>Exclusion: Missing data
[65]	620 (487)	37.2 ± 10.3 vegans,<br>32.7 ± 9.5 vegetarians,<br>34.6 ± 10.8 omnivores	Inclusion: 25–60 years, sampled from social media websites<br>Exclusion: Not healthy (debilitating chronic diseases)
[66]	405 (270)	19.9 ± 1.6	Inclusion: Students from the University of Otago<br>Exclusion: Missing data, dropped out
[67]	9,931 (5,243) at baseline	44.2 ± 18.4 at baseline	Inclusion: ≥ 18 years<br>Exclusion: Living on Indian Reserves or Crown Lands, residents of health institutions, full-time members of the Canadian Forces Bases, living in some remote areas in Ontario and Quebec
[68]	3,594 (2,009)	70 ± 0.3 (SE) [1]	Inclusion: ≥ 60 years, identifying as Hispanic, non-Hispanic Black, or non-Hispanic Chinese, living in ethnic enclaves or areas

Ref.	Number of participants (females)	Age (mean with SD/ range)	Inclusion criteria/ Exclusion criteria
			characterized by high racial/ethnic concentrations<br>Exclusion: -
[69]	728 (397)	21.5 ± 2.8 for male<br>21.2 ± 3.0 for female	Inclusion criteria: first-year students at Gold Coast Campus of Griffith University<br>Exclusion criteria: -
[70]	20,220 (not specified)	≥ 15 years	Inclusion: ≥ 15 years, living in a private household, registered inhabitants<br>Exclusion: Missing data
[71]	3,706 (2,699)	24.9 ± 8.6	Inclusion: Students from 7 universities in England, Wales and Northern Ireland<br>Exclusion: -
[72]	6,271 (6,271)	55.5 ± 1.5	Inclusion: ≥ 18 years, women<br>Exclusion: Missing data
[73]	201 (201)	58.5 ± 11.4	Inclusion: ≥ 18 years old, women, undergoing a clinically indicated coronary angiogram for suspected myocardial ischemia<br>Exclusion: Major comorbidity compromising follow-up, pregnancy, contraindication to provocative diagnostic testing, cardiomyopathy, New York Heart Association class IV heart failure, recent myocardial infarction or revascularization procedure, significant valvular or congenital heart disease, language barrier
[37]	196 (196)	38.3 ± 7.6	Inclusion: BMI ≥ 25kg/m^2, waist circumference ≥ 88 cm, 25–50 years, able to read and speak English, not pregnant, able to participate in moderate to vigorous physical activity, no affirmative responses on the Physical Activity Readiness Questionnaire (PAR-Q), blood pressure <140/90, no impairments that would prohibit participation in data collection, not insulin-dependent diabetes, non-institutional residence<br>Exclusion: Medical contraindications, missing data
[74]	4,215 (1,060)	Not specified for all participants combined	Inclusion: UK civil servants (government employees), 35–55 years at baseline<br>Exclusion: Previous use of anti-depressive drugs, missing data, non-respondent at phase 9
[75]	1,512 (911)	30–75	Inclusion: 30–75 years<br>Exclusion: Pregnant, those judged by the researcher and/or the doctor to be too unwell to participate (patients with terminal illnesses or who had acute emergencies requiring hospital admission), not Roman Catholic or Hindu
[76]	296,121 (not specified)	Not specified for all participants combined	Inclusion: ≥ 12 years<br>Exclusion: Living on Indian Reserves or Crown Lands, residents of in prisons or health care facilities, full-time members of the Canadian Forces, living in certain remote areas
[77]	10,986 (5,840)	18–79 years	Inclusion: ≥ 18 years<br>Exclusion: -
[78]	986 (571)	Not specified for all participants combined	Inclusion: ≥ 70 years, living in the Tsurugaya area of Sendai<br>Exclusion: Missing data, history of cancer, cognitive dysfunction
[79]	9,549 (not specified)	38.4 ± 15.2 for low stress group<br>39.5 ± 16.2 for high stress group	Inclusion criteria: ≥ 18 years, having dietary and anthropometric information, plasma glucose, lipid profile, etc.<br>Exclusion criteria: Previous chronic diseases, taking medications
[80]	281 (153)	19.9 ± 1.2	Inclusion: ≥ 18 years, undergraduate students<br>Exclusion: History of eating disorders
[81]	Approx. 80,000 (not specified)	Not specified	Inclusion criteria: -<br>Exclusion criteria: -

Ref.	Number of participants (females)	Age (mean with SD/ range)	Inclusion criteria/ Exclusion criteria
[82]	97 (69)	18–70 years	Inclusion: > 18 years, members of the Mood Disorders Association of British Columbia (MDABC), residents of lower mainland of British Columbia<br>Exclusion: Individuals with conditions associated with psychotic symptoms, dementia, thyroid dysfunction, or neuro-degeneration
[83]	278 (173)	70.4 ± 6.0	Inclusion: Patients who met DSM-IV diagnostic criteria for major depressive disorder at baseline (studied group) or participants recruited from the community (control group), ≥ 60 years, could speak and write English<br>Exclusion: Concurrent diagnosis of a psychiatric or neurological illness, significant cognitive impairment (Mini-Mental State Examination score of <24 out of 30), severe depression symptomatology (evaluated by the treating psychiatrist) (studied group) or no depression diagnosis (control group), missing data, participants with a total energy intake of < 500 or > 5000 kcal/d
[84]	1,609 (682)	Not specified for all participants combined	Inclusion: ≥ 65 years<br>Exclusion: Depressive symptoms (CES-D score > 10) at baseline, failed to complete the 2003 survey, died
[36]	104 (21)	67.6 ± 9.3	Inclusion: Patients of a teaching hospital in Taipei, metabolic syndrome prior to the survey, based on the criteria of the Department of Health in Taiwan, 3 month to 10 years since CABG surgery, ability to read Chinese<br>Exclusion: Psychiatric diagnosis
[85]	139 (103)	55.3 ± 15.5	Inclusion: ≥ 18 years, living in Hawaii<br>Exclusion: Non-English speakers
[86]	3,714 (2,035)	47.4 ± 10.8 for male<br>46.5 ± 11.1 for female	Inclusion: 25–64 years, registered in Finland<br>Exclusion: BMI <18.5 kg/m^2
[87]	1,190 (637)	75.0 ± 7.0 for male<br>73.0 ± 7.0 for female	Inclusion: ≥ 65 years, from Greek islands and Cyprus<br>Exclusion: Residing in assisted-living centers, clinical history of cardiovascular disease or cancer
[88]	6,803 (3,607)	28.6 ± 0.2 for male<br>28.5 ± 0.2 for female	Inclusion: 17–39 years at interview<br>Exclusion: Participants with a total energy intake of < 500 or > 5000 kcal/d, pregnant or breastfeeding
[89]	1,839 (1,194)	20.6 ± 2.3	Inclusion: First-year undergraduate students<br>Exclusion: Missing data
[90]	1,781 (1,781)	39.2 ± 4.5 for single mothers<br>40.1 ± 4.3 for married/ cohabiting mothers	Inclusion: Mother of child born in Sweden in 1988 and 1989, both she and father of child born in a Scandinavian country, living in Sweden and alive during 1999<br>Exclusion: Missing data, both she and father of child overweight
[91]	773 (NA) at baseline<br>115 (NA) in 2000	72.1 ± 5.2 at baseline<br>84.0 ± 3.5 in 2000	Inclusion criteria: Zutphen Study subjects, men, 64–84 years at baseline<br>Exclusion criteria: Missing data, died
[92]	2,541 (1,071)	20.4	Inclusion: College students (3 and 4 year college)<br>Exclusion: -
[93]	8,690 (not specified)	31	Inclusion criteria: Born in Northern Finland in 1966, alive at the age of 31 years<br>Exclusion criteria: -
[94]	6,243 (4,991)	40–60 years	Inclusion: 40, 45, 50, 55 and 60–year-old women and men, employed by City of Helsinki<br>Exclusion: -
[95]	418 (not specified)	Not specified	Inclusion: Swansea electoral register lists of voters over the age of 18 years<br>Exclusion: Missing data (age, sex or occupation)

[1] Data provided by authors on request; SD – standard deviation; SE – standard error.

Supplementary Table 4. Detailed results of the quality assessment based on the total score for the Newcastle-Ottawa Scale for categories of selection, comparability and exposure/outcome.

Ref.	Scores for categories			Quality	
	**Selection**	**Comparability**	**Exposure/outcome**	**Total score***	**Risk of bias**
[38]	3	2	0	5	High
[39]	3	2	0	5	High
[40]	2	2	2	6	High
[41]	2	2	1	5	High
[42]	2	2	1	5	High
[43]	2	2	2	6	High
[35]	1	2	2	5	High
[44]	3	2	0	5	High
[45]	1	2	0	3	Very high
[46]	4	2	1	7	Low
[47]	2	2	2	6	High
[48]	0	2	0	2	Very high
[49]	2	2	1	5	High
[50]	3	0	1	4	High
[51]	2	2	0	4	High
[52]	3	2	1	6	High
[53]	0	2	1	3	Very high
[54]	3	2	2	7	Low
[55]	2	2	1	5	High
[56]	4	2	2	8	Low
[57]	2	2	1	5	High
[58]	2	2	1	5	High
[59]	2	2	1	5	High
[60]	1	2	0	3	Very high
[61]	3	2	1	6	High
[62]	3	2	2	7	Low
[63]	0	2	1	3	Very high
[64]	2	2	1	5	High
[65]	0	1	1	2	Very high
[66]	0	2	2	4	High
[67]	3	2	3	8	Low
[68]	3	2	0	5	High
[69]	1	2	1	4	High
[70]	3	2	0	5	High
[71]	1	2	2	5	High
[72]	2	2	2	6	High
[73]	2	2	2	6	High

Ref.	Scores for categories			Quality	
	Selection	Comparability	Exposure/outcome	Total score*	Risk of bias
[37]	3	2	1	6	High
[74]	2	2	2	6	High
[75]	3	2	0	5	High
[76]	2	2	1	5	High
[77]	3	2	1	6	High
[78]	3	2	1	6	High
[79]	3	2	0	5	High
[80]	0	2	1	3	Very high
[81]	2	2	0	4	High
[82]	2	0	2	4	High
[83]	3	2	1	6	High
[84]	3	2	2	7	Low
[36]	2	0	0	2	Very high
[85]	1	2	1	4	High
[86]	3	2	1	6	High
[87]	3	2	1	6	High
[88]	4	2	0	6	High
[89]	1	2	1	4	High
[90]	3	2	1	6	High
[91]	3	2	2	7	Low
[92]	3	2	0	5	High
[93]	3	2	0	7	Low
[94]	2	2	1	5	High
[95]	3	0	1	4	High

* total score for the Newcastle-Ottawa Scale (NOS) is attributed to a following categories: very high risk of bias (0-3 NOS points), high risk of bias (4-6 NOS points), and low risk of bias (7-9 NOS points) [23].

© 2020 by the authors. Licensee MDPI, Basel, Switzerland. This article is an open access article distributed under the terms and conditions of the Creative Commons Attribution (CC BY) license (http://creativecommons.org/licenses/by/4.0/).
